# Tracing the dynamics of gene transcripts after organismal death

**DOI:** 10.1098/rsob.160267

**Published:** 2017-01-25

**Authors:** Alex E. Pozhitkov, Rafik Neme, Tomislav Domazet-Lošo, Brian G. Leroux, Shivani Soni, Diethard Tautz, Peter A. Noble

**Affiliations:** 1 Department of Oral Health Sciences, University of Washington, PO Box 357444, Seattle, WA 98195, USA; 2 Department of Periodontics, University of Washington, PO Box 357444, Seattle, WA 98195, USA; 3 Max Planck Institute for Evolutionary Biology, August-Thienemann-Strasse 2, 24306 Ploen, Germany; 4 Laboratory of Evolutionary Genetics, Division of Molecular Biology, Ruđer Bošković Institute, 10002 Zagreb, Croatia; 5 Catholic University of Croatia, Ilica 242, Zagreb, Croatia; 6 Department of Biological Sciences, Alabama State University, Montgomery, AL 36101-0271, USA; 7 PhD Program in Microbiology, Alabama State University, Montgomery, AL 36101-0271, USA

**Keywords:** postmortem transcriptome, Gene Meters, cancer, developmental control, transplantology, forensic science

## Abstract

In life, genetic and epigenetic networks precisely coordinate the expression of genes—but in death, it is not known if gene expression diminishes gradually or abruptly stops or if specific genes and pathways are involved. We studied this by identifying mRNA transcripts that apparently increase in relative abundance after death, assessing their functions, and comparing their abundance profiles through postmortem time in two species, mouse and zebrafish. We found mRNA transcript profiles of 1063 genes became significantly more abundant after death of healthy adult animals in a time series spanning up to 96 h postmortem. Ordination plots revealed non-random patterns in the profiles by time. While most of these transcript levels increased within 0.5 h postmortem, some increased only at 24 and 48 h postmortem. Functional characterization of the most abundant transcripts revealed the following categories: stress, immunity, inflammation, apoptosis, transport, development, epigenetic regulation and cancer. The data suggest a step-wise shutdown occurs in organismal death that is manifested by the apparent increase of certain transcripts with various abundance maxima and durations.

## 1. Introduction

A healthy adult vertebrate is a complex biological system capable of highly elaborate functions such as the ability to move, communicate and sense the environment—all at the same time. These functions are tightly regulated by genetic and epigenetic networks through multiple feedback loops that precisely coordinate the expression of thousands of genes at the right time, in the right place and in the right level [1]. Together, these networks maintain homeostasis and thus sustain ‘life’ of a biological system.

While much is known about gene expression circuits in life, there is a paucity of information about what happens to these circuits after organismal death. For example, it is not well known whether gene expression diminishes gradually or abruptly stops in death—nor whether specific gene transcripts increase in abundance in death*.* In organismal ‘death’, defined here as the cessation of the highly elaborate system functions in vertebrates, we conjecture that there is a gradual disengagement and loss of global regulatory networks as well as the activation of regulatory genes involved in survival and stress compensation. To test this, we examined the global postmortem abundances of mRNAs in two model organisms: the zebrafish, *Danio rerio,* and the house mouse, *Mus musculus.* The purpose of the research was to investigate the ‘unwinding of the clock’ by identifying mRNA transcripts that increase in abundance with postmortem time and assessing their functions based on the primary literature. The biological systems investigated in this study are different from those examined in other studies, such as individual dead and/or injured cells in live organisms, i.e. apoptosis and necrosis (reviewed in [2–5]). In contrast to previous studies, the abundances of mRNA transcripts from the entire *D. rerio* body, and the brains and livers of *M. musculus* were assessed through postmortem time. The mRNA transcripts were measured using the ‘Gene Meter’ approach that precisely reports transcript abundances based on a calibration curve for each microarray probe [6–9].

## 2. Material and methods

### 2.1. Induced death and postmortem incubation

#### 2.1.1. Zebrafish

Forty-four female *Danio rerio* were transferred from several flow-through aquaria kept at 28°C to a glass beaker containing 1 l of aquarium water. Four individuals were immediately taken out, snap frozen in liquid nitrogen and stored in Falcon tubes at −80°C (two zebrafish per tube). These samples were designated as the first set of live controls. A second set of live controls was immersed in an open cylinder (described below). Two sets of live controls were used to determine whether putting the zebrafish back into their native environment had any effects on gene expression (we later discovered no significant effects).

The rest of the zebrafish were subjected to sudden death by immersion in a ‘kill’ chamber. The chamber consisted of an 8 l styrofoam container filled with chilled ice water. To synchronize the death of the rest of the zebrafish, they were transferred to an open cylinder with a mesh-covered bottom and the cylinder was immersed into the kill chamber. After 20–30 s of immersion, four zebrafish were retrieved from the chamber, snap frozen in liquid nitrogen and stored at −80°C (two zebrafish per Falcon tube). These samples were designated as the second set of live controls. The remaining zebrafish were kept in the kill chamber for 5 min and then the cylinder was transferred to a flow-through aquarium kept at 28°C so that they were returned to their native environment.

Postmortem sampling of the zebrafish occurred at: time 0, 15 min, 30 min, 1 h, 4 h, 8 h, 12 h, 24 h, 48 h and 96 h. For each sampling time, four expired zebrafish were retrieved from the cylinder, snap frozen in liquid nitrogen and stored at −80°C in Falcon tubes (two zebrafish to a tube). One zebrafish sample was lost, but extraction volumes were adjusted to one individual.

#### 2.1.2. Mouse

The mouse strain C57BL/6JRj (Janvier SAS, France) was used for our experiments. The mice were 20-week old males of approximately the same weight. The mice were highly inbred and were expected to have a homogeneous genetic background. Prior to euthanasia, the mice were kept at room temperature and were given *ad libitum* access to food and water. Each mouse was euthanized by cervical dislocation and placed in an individual plastic bag with holes to allow air/gas exchange. The bagged carcasses were kept at room temperature in a large, open polystyrene container. Sampling of the deceased mice began at 0 h (postmortem time zero) and continued at 30 min, 1 h, 6 h, 12 h, 24 h and 48 h postmortem. At each sample time, three mice were sampled (except for 48 h when two mice were sampled) and the entire brain (plus stem) and two portions of the liver were extracted from each mouse. For liver samples, clippings were taken from the foremost and rightmost lobes of the liver. The brain and liver samples were snap frozen in liquid nitrogen and stored individually in Falcon tubes at −80°C.

### 2.2. RNA extraction, labelling, hybridization and DNA microarrays

The number of individuals was 43 for zebrafish and 20 for mice. Samples from two fish were pooled for analysis, resulting in two replicate measurements at each time point. The number of replicated measurements for mice was three at each of the first six time points and two at 48 h. Thus, the total number of samples analysed was 22 for zebrafish and 20 for mice. For the zebrafish, samples were mixed with 20 ml of Trizol and homogenized using a TissueLyzer (Qiagen). For the mice, 100 mg of brain or liver samples were mixed with 1 ml of Trizol and homogenized. One millilitre of the emulsion from each sample was put into a fresh 1.5 ml centrifuge tube for RNA extraction and the rest was frozen at −80°C.

RNA was extracted by adding 200 µl of chloroform, vortexing the sample and incubating it at 25°C for 3 min. After centrifugation (15 min at 12 000*g* at 4°C), the supernatant (approx. 350 µl) was transferred to a fresh 1.5 ml tube containing an equal volume of 70% ethanol. The tube was vortexed, centrifuged and purified following the procedures outlined in the PureLink RNA Mini Kit (Life Technologies, USA).

The isolated RNA, 400 ng per sample, was labelled, purified and hybridized according to the One-Color Microarray-based Gene Expression Analysis (Quick Amp Labeling) with Tecan HS Pro Hybridization kit (Agilent Technologies). For the zebrafish, the labelled RNA was hybridized to the Zebrafish (v2) Gene Expression Microarray (Design ID 019161). For the mouse, the labelled RNA was hybridized to the SurePrint G3 Mouse GE 8×60K Microarray Design ID 028005 (Agilent Technologies). The microarrays were loaded with 1.65 µg of labelled cRNA for each postmortem sample.

### 2.3. Microarray calibration

Oligonucleotide (60 nt) probes on the zebrafish and mouse microarrays were calibrated using pooled labelled cRNA of all zebrafish and all mouse postmortem samples, respectively. The dilution series for the Zebrafish array was created using the following concentrations of labelled cRNA: 0.41, 0.83, 1.66, 1.66, 1.66, 3.29, 6.60 and 8.26 µg. The dilution series for the Mouse arrays was created using the following concentrations of labelled cRNA: 0.17, 0.33, 0.66, 1.32, 2.64, 5.28, 7.92 and 10.40 µg. Calibration involved plotting the signal intensities of the probes against a dilution factor and determining the isotherm model (e.g. Freundlich and/or Langmuir) that best fit the relationship between signal intensities and gene abundances.

**Figure 1.**
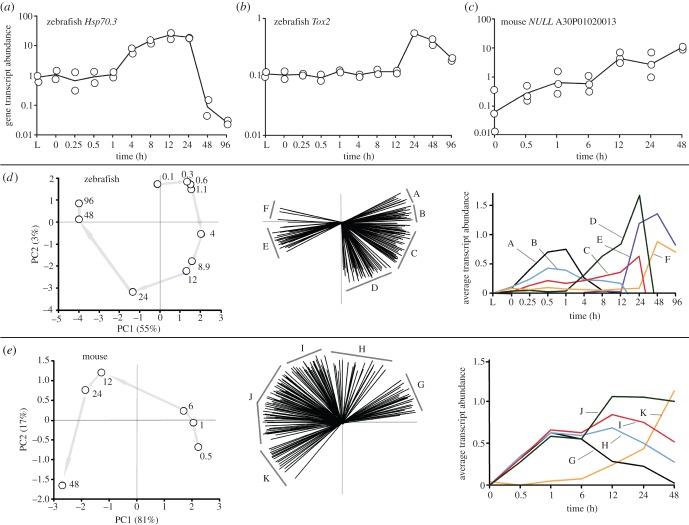
Transcriptional profiles of representative genes (arb. units), ordination plots based on transcript abundances by postmortem time (h) with corresponding transcript contributions (biplots), and averaged transcript abundances by group. (*a–c*) Transcriptional profiles of (*a*) the *Hsp70.3* gene, (*b*) the *Tox2* gene and (*c*) a non-annotated transcript ‘NULL’ (i.e. no annotation, probe number shown) gene as a function of postmortem time. (*d,e*) Ordination plots of the (*d*) zebrafish and (*e*) mouse were based on all gene transcript profiles found to have a significantly increased abundance. Gene transcripts in the biplots were arbitrarily assigned alphabetical groups based on their positions in the ordination. The average transcript abundances for each group are shown.

Consider zebrafish gene transcripts targeted by A_15_P110618 (which happens to be one of the transcriptional profiles of gene *Hsp70.3* shown in figure 1*a*). External file FishProbesParameters.txt shows that a Freundlich model best fit the dilution curve with *R*^2^ = 0.99. The equation for this probe is the following:
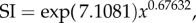
where SI is the observed average signal intensity for the dilution *x.* The transcript abundance *G* was calculated by inverting this equation. For each probe signal intensity at postmortem time, SI*_t_,* the gene abundance *G* *=* (SI*_t_*/exp(7.1081))^1/0.67632^. Specifically, consider two biological replicates of 15 min postmortem zebrafish, the signal intensities of the probe A_15_P110618 are 770.5 and 576, which translates into the abundances 0.50 and 0.33 arbitrary units (arb. units), respectively. The target abundances were further converted to log10 and are shown in external file Fish_log10_AllProfiles.txt.

Further details of the calibration protocols used to calculate RNA transcript relative abundances are provided elsewhere [6,7].

### 2.4. Statistical analysis

Abundance levels were log-transformed for analysis to stabilize the variance. A one-sided Dunnett's *T*-statistic was applied to test for increase at one or more postmortem times compared to live control (fish) or time 0 (mouse). A bootstrap procedure with 10^9^ simulations was used to determine the critical value for the Dunnett's statistics in order to accommodate departures from parametric assumptions and to account for multiplicity of testing. The transcript profile for each gene was centred by subtracting the mean values at each postmortem time point to create ‘null’ profiles. Bootstrap samples of the null profiles were generated to determine the 95th percentile of the maximum (over all genes) of the Dunnett's statistics. A transcript was considered to have a significantly increased abundance when one or more points had Dunnett's *T*-values larger than the 95th percentile. The corresponding genes were retained for further analyses.

Orthogonal transformation of the abundances to their principal components (PCs) was conducted, and the results were graphed on a two-dimensional ordination plot. The *m ×* *n* matrix of abundances (sampling times by number of gene transcripts), which is 10 × 548 for zebrafish and 7 × 515 for mouse, was used to produce an *m* × *m* matrix *D* of Euclidean distances between all pairs of sampling times. Principal component analysis (PCA) was performed on the matrix of distances, *D*. To investigate and visualize differences between the sampling times, a scatterplot of the first two principal components (PC1 and PC2) was created. To establish relative contributions of the gene transcripts, the projection of each sampling time onto the (PC1 and PC2) plane was calculated and those gene transcripts with high correlations (greater than or equal to 0.70) between abundances and either component (PC1 or PC2) were displayed as a biplot.

### 2.5. Gene annotation and functional categorization

Microarray probe sequences were individually annotated by performing a BLASTN search of the zebrafish and mouse NCBI databases (February 2015). The gene annotations were retained if the bit score was greater than or equal to 100 and the annotations were in the correct 5′–3′ orientation. Transcription factors, transcriptional regulators and cell signalling components (e.g. receptors, enzymes and messengers) were identified as global regulatory genes. The rest were considered response genes.

Functional categorizations were performed by querying the annotated gene transcripts in the primary literature and using UniProt (www.uniprot.org). Genes not functionally categorized to their native organism (zebrafish or mouse) were categorized to genes of phylogenetically related organisms (e.g. human). Cancer-related genes were identified using a previously constructed database (see Additional File 1: table S1 in [10]).

## 3. Results

Extracting the total mRNA, calibrating the microarray probes, and determining the transcript abundances at each postmortem sampling time produced a fine-grain series of transcriptome data for the zebrafish and the mouse. Approximately 84.3% (36 811 of 43 663) zebrafish probes and 67.1% (37 368 of 55 681) mouse probes were found to provide suitable dose–response curves for calibration (electronic supplementary material, files S1–S7; http://dx.doi.org/10.5061/dryad.hv223).

**Figure 2.**
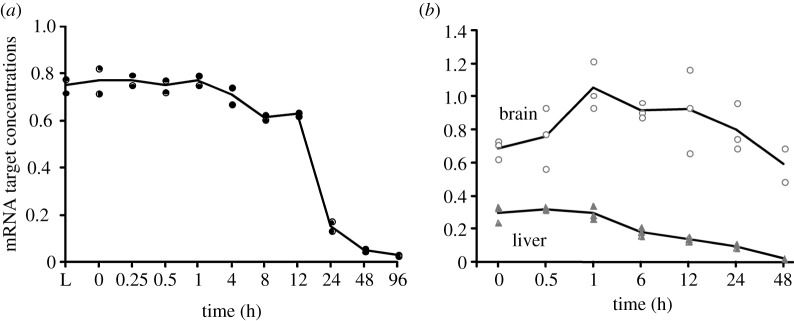
Total mRNA abundance (arbitrary units, arb. units) by postmortem time determined using all calibrated microarray probes: (*a*) extracted from whole zebrafish, (*b*) extracted from the brain and liver tissues of whole mice. Each datum point represents the mRNA from two individuals in the zebrafish and a single individual in the mouse.

Figure 2 shows the sum of all transcript abundances calculated from the calibrated probes in dependence of postmortem time. In general, the sum of all abundances decreased with time, which means that less transcript targets hybridized to the microarray probes. In the zebrafish, mRNA decreased abruptly at 12 h postmortem (figure 2*a*), while for the mouse brain (figure 2*b*), mRNA increased in the first hour and then gradually decreased. For the mouse liver, mRNA gradually decreased with postmortem time. The fact that total mRNA shown in figure 2*a,b* mirrors the electrophoresis patterns shown in the electronic supplementary material, figures S1 and S2 (ignoring the 28S and 18S rRNA bands) indicates a general agreement of the Gene Meter approach to the gel-based approach (i.e. Agilent Bioanalyzer). Hence, mRNA abundances depend on the organism (zebrafish, mouse), organ (brain, liver) and postmortem time, which is aligned with previous studies [11–16].

The abundance of a transcript is determined by its rate of synthesis and its rate of degradation [17]. We focused here on transcripts that significantly increased in abundance—relative to live controls—because these genes might be actively transcribed after organismal death despite an overall decrease in total mRNA with time. A transcript was defined as having a significantly increased abundance when at least one time point was statistically higher than that of the control (figure 1*a–c*). It is important to understand that the entire profiles, i.e. 22 data points for the zebrafish and 20 points for the mouse, were subjected to a statistical test to determine significance (see Material and methods). We found 548 zebrafish profiles and 515 mouse profiles had significantly increased transcript abundances.

Based on GenBank gene annotations, we found that, among the transcripts with significantly increased abundances, for the zebrafish 291 were protein-coding genes (53%) and 257 non-annotated mRNA (47%), and for the mouse 324 were known protein-coding genes (63%), 190 non-annotated mRNA (37%) and one an Agilent control sequence of unknown composition. Hence, in the zebrafish and mouse, about 58% of the total genes with significant transcript abundances are known and the rest (42%) are putatively non-annotated RNA.

Examples of genes yielding transcripts that significantly increased in abundance with postmortem time are: the Heat shock protein (*Hsp70.3)* gene, the Thymocyte selection-associated high mobility group box 2 (*Tox2*) gene, and an unknown (*NULL*) gene (figure 1*a–c*). While the *Hsp70.3* transcript abundance increased after 1 h postmortem to reach a maximum at 12 h, the *Tox2* transcript increased after 12 h postmortem to reach a maximum at 24 h, and the *NULL* transcript consistently increased with postmortem time. These figures provide typical examples of transcript profiles and depict the high reproducibility of the sample replicates as well as the quality of output obtained by the Gene Meter approach.

### 3.1. Non-random patterns in transcript profiles

Ordination plots of the transcript profiles that had significantly increased abundances revealed prominent differences with postmortem time (figure 1*d,e*), suggesting the increases in transcript abundances of genes followed a discernable (non-random) pattern in both organisms. The biplots showed that 203 zebrafish transcript profiles and 226 mouse profiles significantly contributed to the ordinations. To identify patterns in the transcript profiles, we assigned them to groups based on their position in the biplots. Six profile groups were assigned for the zebrafish (A to F) and five groups (G to K) were assigned for the mouse. Determination of the average gene transcript abundances by group revealed differences in the shapes of the averaged profiles, particularly the timing and magnitude of peak abundances, which accounted for the positioning of data points in the ordinations.

Genes coding for global regulatory functions were examined separately from others (i.e. response genes). Combined results show that about 33% of the genes in the ordination plots were involved in global regulation, with 14% of these encoding transcription factors/transcriptional regulators and 19% encoding cell signalling proteins such as enzymes, messengers and receptors (electronic supplementary material, table S3). The response genes accounted for 67% of the total.

The genes were assigned to 22 categories (electronic supplementary material, File S8) with some genes having multiple categorizations. For example, the Eukaryotic translation initiation factor 3 Subunit J-B (*Eif3j2*) gene was assigned to protein synthesis and cancer categories [18].

Genes in the following functional categories were investigated: stress, immunity, inflammation, apoptosis, solute/ion/protein transport, embryonic development, epigenetic regulation and cancer. We focused on these categories because they were common to both organisms, they contained multiple genes and they might provide possible explanations for the postmortem increases in transcript abundances (e.g. epigenetic gene regulation, embryonic development, cancer). The transcriptional profiles were plotted by category and each profile was ordered by the timing of the increased abundance and peak abundances. This allowed comparisons of transcript dynamics as a function of postmortem time for both organisms. For each category, we provided the name and function of the gene and compared transcript dynamics within and between the organisms.

### 3.2. Stress response

In organismal death, transcripts from stress response genes were anticipated to significantly increase in abundance because these genes are activated in life to cope with perturbations, recover homeostasis [19] and stabilize the cytoskeleton [20]. The stress response genes were assigned to three groups: heat shock protein (*Hsp*), hypoxia-related and ‘other’ responses such as oxidative stress.

#### 3.2.1. Hsp

**Figure 3.**
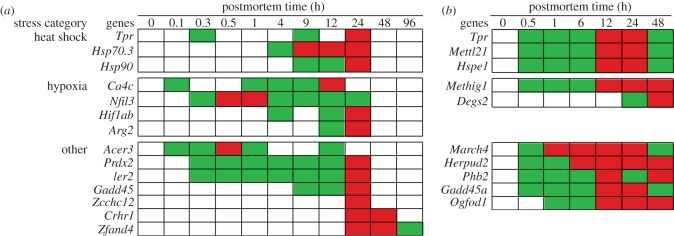
Increased abundance of stress response gene transcripts by postmortem time (h) and stress category: (*a*) zebrafish and (*b*) mouse. Green, intermediate value; red, maximum value.

In the zebrafish, *Hsp* gene transcripts that significantly increased in abundance included: Translocated promoter region (*Tpr*), *Hsp70.3* and *Hsp90* (figure 3). The *Tpr* gene encodes a protein that facilitates the export of the *Hsp* mRNA across the nuclear membrane [21] and has been implicated in chromatin organization, regulation of transcription, mitosis [22] and controlling cellular senescence [23]. The *Hsp70.3* and *Hsp90* genes encode proteins that control the level of intracellular calcium [24], assist with protein folding and aid in protein degradation [25].

In the mouse, the *Hsp* gene transcripts included: *Tpr*, *Hsp*-associated methyltransferase (*Mettl21*) and Heat shock protein 1 (*Hspe1*) (figure 3). The *Mettl21* gene encodes a protein modulating *Hsp* functions [26]. The *Hspe1* gene encodes a chaperonin protein that assists with protein folding in the mitochondria [27].

The timing and duration of the *Hsp* transcript abundances varied by organism. In general, the increase in transcript abundance of *Hsp* genes occurred much later in the zebrafish than the mouse (4 h versus 0.5 h postmortem, respectively). There were also differences in transcript abundance maxima of *Hsp* genes since they reached maxima at 9–24 h in the zebrafish, while they reached maxima at 12–24 h in the mouse. Previous studies have examined the increase of *Hsp70.3* transcripts with time in live serum-stimulated human cell lines [28]. In both the zebrafish and human cell lines (figure 1*a*), the *Hsp70.3* gene transcript reached a maximum abundance at approximately 12 h indicating the same reactions occur in life and death.

#### 3.2.2. Hypoxia

In the zebrafish, hypoxia-related gene transcripts that significantly increased in abundance included: Carbonic anhydrase 4 (*Ca4c*), Nuclear factor (NF) interleukin-3 (*Nfil3*), Hypoxia-inducible factor 1-alpha (*Hiflab*) and Arginase-2 (*Arg2*) (figure 3). The Carbonic anhydrase 4 (*Ca4c*) gene encodes an enzyme that converts carbon dioxide into bicarbonate in response to anoxic conditions [29]. The *Nfil3* gene encodes a protein that suppresses hypoxia-induced apoptosis [30] and activates immune responses [31]. The *Hiflab* gene encodes a transcription factor that prepares cells for decreased oxygen [32]. The *Arg2* gene encodes an enzyme that catalyses the conversion of arginine to urea under hypoxic conditions [33]. Of note, the accumulation of urea presumably triggered the increase of *Slc14a2* gene transcripts at 24 h, as reported in the Transport Section (below).

In the mouse, the hypoxia-related gene transcripts that significantly increased in abundance included: Methyltransferase hypoxia-inducible domain (*Methig1*) and Sphingolipid delta-desaturase (*Degs2*) (figure 3). The *Methig1* gene encodes methyltransferase that presumably is involved in gene regulation [34]*.* The *Degs2* gene encodes a protein that acts as an oxygen sensor and regulates ceramide metabolism [35]. Ceramides are waxy lipid molecules in cellular membranes that regulate cell-growth, death, senescence, adhesion, migration, inflammation, angiogenesis and intracellular trafficking [36].

The increased abundance of *Ca4c* transcripts in the zebrafish putatively indicates a build up of carbon dioxide 0.1–1 h postmortem in the zebrafish presumably due to lack of blood circulation. The increased abundance of the *Nfil3* transcripts in the zebrafish and *Methig1* transcripts in the mouse suggests hypoxic conditions exist within 0.5 h postmortem in both organisms. The increased abundance of the other hypoxia gene transcripts varied with postmortem time, with increases of *Hiflab, Arg2* and *Degs2* transcripts at 4 h, 12 h and 24 h, respectively.

#### 3.2.3. Other stress responses

In the zebrafish, gene transcripts that significantly increased in abundance included: Alkaline ceramidase 3 (*Acer3*), Peroxirodoxin 2 (*Prdx2*), Immediate early (*Ier2*), Growth arrest and DNA-damage-inducible protein (*Gadd45a*), Zinc finger CCH domain-containing 12 (*Zcchc12*), Corticotropin-releasing hormone receptor 1 (*Crhr1*) and Zinc finger AN1-type domain 4 (*Zfand4*) (figure 3). The *Acer3* gene encodes a stress sensor protein that mediates cell-growth arrest and apoptosis [37]. The *Prdx2* gene encodes an antioxidant enzyme that controls peroxide levels in cells [38] and triggers production of *Tnfa* proteins that induce inflammation [39]. The *Ier2* gene encodes a transcription factor involved in stress response [40]. The *Gadd45a* gene encodes a stress protein sensor that stops the cell cycle [41], modulates cell death and survival, and is part of the signalling networks in immune cells [42]. The *Zcchc12* gene encodes a protein involved in stress response in the brain [43]. The *Crhr1* and *Zfand4* genes encode stress proteins [44,45].

While the *Acer3, Prdx2* and *Ier2* transcripts increased within 0.3 h postmortem, indicating a changed physiological state, the *Gadd45a* transcript increased at 9 h and the other transcripts (*Zcchc12, Crhr1 and Zfand4*) increased at 24 h postmortem.

In the mouse, gene transcripts that significantly increased in abundance included: Membrane-associated RING-CH 4 (*March4*), Homocysteine-responsive endoplasmic reticulum-resident ubiquitin-like domain member 2 (*Herpud2*), Prohibitin-2 (*Phb2*), *Gadd45a* and Two-oxoglutarate and iron-dependent oxygenase domain-containing 1 (*Ogfod1*) (figure 3). The *March4* gene encodes an immunologically-active stress response protein [46]. The *Herpud2* gene encodes a protein that senses the accumulation of unfolded proteins in the endoplasmic reticulum [47]. The *Phb2* gene encodes a cell surface receptor that responds to mitochondrial stress [48]. The *Ogfod1* gene encodes a stress-sensing protein [49].

Note that the stress gene transcripts in the mouse all increased within 1 h postmortem and remained at high abundance for 48 h.

#### 3.2.4. Summary of stress response

In both organisms, organismal death increased the abundance of heat shock, hypoxia and ‘other stress’ gene transcripts, which varied in their timing and duration within and between organisms. Consider, for example, the *Tpr* and *Gadd45a* genes, which were common to both organisms. While the transcript abundance for the *Tpr* gene significantly increased within 0.5 h postmortem in both organisms, the transcript abundance for the *Gadd45a* gene increased at 9 h in the zebrafish and 0.5 h in the mouse. In addition, the transcriptional profile of the *Tpr* gene was more variable in the zebrafish than that of the mouse since the transcripts increased in abundance at 0.3 h, 9 h and 24 h postmortem, which suggests that they might be regulated through a feedback loop. By contrast, the transcriptional profile of *Tpr* gene in the mouse increased at 0.5 h and peaked at 12 and 24 h postmortem.

Taken together, the significant increase in transcript abundance of stress genes in both organisms is presumably to compensate for a loss of homeostasis.

### 3.3. Innate and adaptive immune responses

In organismal death, an increase of immune response gene transcripts was anticipated since vertebrates have evolved ways to protect the host against infection in life, even under absolutely sterile conditions [50]. Inflammation genes were excluded from this section (even though they are innate immune genes) because we examined them in a separate section (below).

**Figure 4.**
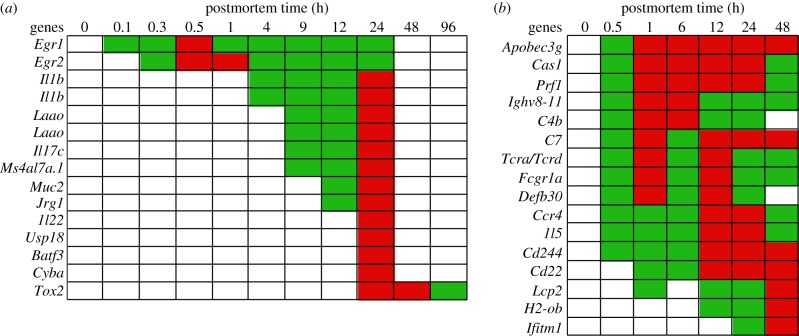
Abundance of immunity gene transcripts by postmortem time (h): (*a*) zebrafish and (*b*) mouse. Green, intermediate value; red, maximum value. Some transcripts were represented by two different probes (e.g. *Il1b*, *Laao*).

In the zebrafish, gene transcripts that significantly increased in abundance included: Early growth response-1 and -2 (*Egr1, Egr2*), Interleukin-1b (*Il1b*), l-amino acid oxidase (*Laao*), Interleukin-17c (*Il17c*), Membrane-spanning 4-domains subfamily A member 17A.1 (*Ms4a17.a1*), Mucin-2 (*Muc2*), Immunoresponsive gene 1 (*Irg1*), Interleukin-22 (*Il22*), Ubl carboxyl-terminal hydrolase 18 (*Usp18*), ATF-like 3 (*Batf3*), Cytochrome b-245 light chain (*Cyba*) and Thymocyte selection-associated high mobility group box protein family member 2 (*Tox2*) (figure 4). The *Egr1* and *Egr2* genes encode proteins that regulate B- and T-cell functions in adaptive immunity [51,52]. The *Il1b* gene encodes an interleukin that kills bacterial cells through the recruitment of other antimicrobial molecules [53]. The *Laao* gene encodes an oxidase involved in innate immunity [54]. The *Il17c* and *Il22* genes encode interleukins that work synergically to produce antibacterial peptides [55]. The *Ms4a17.a1* gene encodes a protein involved in adaptive immunity [56]. The *Muc2* gene encodes a protein that protects the intestinal epithelium from pathogenic bacteria [57]. The *Irg1* gene encodes an enzyme that produces itaconic acid, which has antimicrobial properties [58]. The *Usp18* gene encodes a protease that plays a role in adaptive immunity [59]. The *Batf3* gene encodes a transcription factor that activates genes involved in adaptive immunity [60]. The *Cyba* gene encodes an oxidase that is used to kill microorganisms [61]. The *Tox2* gene encodes a transcription factor that regulates natural killer (NK) cells of the innate immune system [62].

Increases of immunity gene transcripts in the zebrafish occurred at different times with varying durations. While transcripts of genes involved in adaptive immunity increased in abundance at 0.1–0.3 h (*Egr*), 9 h (*Ms4a17.a1*) and 24 h (*Usp18, Batf3*) postmortem, transcripts of genes involved in innate immunity increased at 4 h (*Il1b*), 9 h (*Laao, Il17c*), 12 h (*Muc2, Irg1*) and 24 h (*Il22, Cyba,Tox2*), indicating a multi-pronged and progressive approach to deal with injury and the potential of microbial invasion.

In the mouse, gene transcripts that significantly increased in abundance included: Catalytic polypeptide-like 3G (*Apobec3g*), CRISPR-associated endonuclease (*Cas1*), Perforin-1 (*Prf1*), Immunoglobulin heavy variable 8–11 (*Ighv8-11*), C4b-binding protein (*C4b*), Complement component C7 (*C7*), T-cell receptor alpha and delta chain (*Tcra/Tcrd*), High affinity immunoglobulin gamma Fc receptor I (*Fcgr1a*), Defensin (*Defb30*), Chemokine-4 (*Ccr4*), Interleukin-5 (*Il5*), NK cell receptor 2B4 (*Cd244*), Cluster of differentiation-22 (*Cd22*), Lymphocyte cytosolic protein 2 (*Lcp2*), Histocompatibility 2 O region beta locus (*H2ob*) and Interferon-induced transmembrane protein 1 (*Ifitm1*) (figure 4). The *Apobec3g* gene encodes a protein that plays a role in innate anti-viral immunity [63]. The *Cas1* gene encodes a protein involved in regulating the activation of immune systems [64–67]. The *Prf1, C7* and *Defb30* genes encode proteins that kill bacteria by forming pores in plasma membrane of target cells [68–70]. The *Ighv8-11* gene encodes an immunoglobulin of uncertain function. The *C4b* gene encodes a protein involved in the complement system [71]. The *Tcra/Tcrd* genes encode proteins that play a role in the immune response [72]. The *Fcgr1a* gene encodes a protein involved in both innate and adaptive immune responses [73]. The *Ccr4* gene encodes a cytokine that attracts leucocytes to sites of infection [74]. The *Il5* gene encodes an interleukin involved in both innate and adaptive immunity [75,76]. The *Cd244* and *Cd22* genes encode proteins involved in innate immunity [77]. The *Lcp2* gene encodes a signal-transducing adaptor protein involved in T cell development and activation [78]. The *H2ob* gene encodes a protein involved in adaptive immunity. The *Ifitm1* gene encodes a protein that has anti-viral properties [79].

Most of the transcripts of immune response genes increased in abundance within 1 h postmortem in the mouse (*n* = 14 out of 16 genes), indicating a more rapid response than that of the zebrafish.

#### 3.3.1. Summary of immune response

The increase in transcript abundance of immune response genes in both organisms included innate and adaptive immunity components. An interesting phenomenon observed in the mouse (but not zebrafish) was that four genes (*C7*, *Tcra/Tcrd, Fcgr1a* and *Defb30*) reached transcript abundance maxima at two different postmortem times (i.e. 1 h and 12 h) while others reached only one. The variability in the gene transcript abundances suggests possible regulation by feedback loops.

### 3.4. Inflammation response

**Figure 5.**
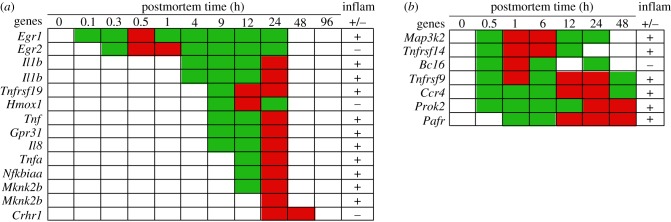
Abundance of inflammation gene transcripts by postmortem time (h): (*a*) zebrafish and (*b*) mouse. Inflammation, pro-, +; anti, −. Green, intermediate value; red, maximum value. The *Il1b* and *Mknk2b* genes were represented by two different probes.

The increased abundance of transcripts of inflammation genes in organismal death was anticipated because inflammation is an innate immunity response to injury. In the zebrafish, inflammation gene transcripts that increased in abundance included: *Egr1, Egr2*, *Il1b*, Tumour necrosis factor receptor (*Tnfrsf19*), Haem oxygenase 1 (*Hmox1*), Tumour necrosis factor (*Tnf*), G-protein receptor (*Gpr31*), Interleukin-8 (*Il8*), Tumour necrosis factor alpha (*Tnfa*), NF kappa B (*Nfkbiaa*), MAP kinase-interacting serine/threonine kinase 2b (*Mknk2b*) and Corticotropin-releasing factor receptor 1 (*Crhr1*) (figure 5). The *Egr1* and *Egr2* genes encode transcription factors that are pro- and anti-inflammatory, respectively [51,52,80]. The *Il1b* gene encodes a pro-inflammatory cytokine that plays a key role in sterile inflammation [81,82]. The *Tnfrsf19* gene encodes a receptor that has pro-inflammatory functions [83]. The *Hmox1* gene encodes an enzyme that has anti-inflammatory functions and is involved in haem catabolism [84,85]. The *Tnf* and *Tnfa* genes encode pro-inflammatory proteins. The *Gpr31* gene encodes a pro-inflammatory protein that activates the NF-κB signalling pathway [86]. The *Il8* gene encodes a cytokine that has pro-inflammatory properties [87]. The *Nfkbiaa* gene encodes a protein that integrates multiple inflammatory signalling pathways including *Tnf* genes [88]. The *Mknk2b* gene encodes a protein kinase that directs cellular responses and is pro-inflammatory [89]. The *Crhr1* gene modulates anti-inflammatory responses [90].

The increased abundance of the pro-inflammatory *Egr1* transcript at 0.1 h was followed by an increase of anti-inflammatory *Egr2* transcript at 0.2 h, suggesting the increase of one transcript was effecting another (figure 5). Similarly, the increased abundance of pro-inflammatory *Il1b* transcript at 4 h postmortem was followed by: increased abundance of pro-inflammatory *Tnfrsf19, Tnf, Gpr31* and *Il8* transcripts and the anti-inflammatory *Hmox1* transcript at 9 h, the increased abundance of pro-inflammatory *Tnfa, Nfkbiaa* and *Mknk2b* transcripts at 12 h, and the increased abundance of anti-inflammatory *Crhr1* transcripts at 24 h. Of note, while none of the pro-inflammatory gene transcripts increased in abundance past 24 h, the anti-inflammatory *Crhr1* gene remained at high abundance at 48 h. It should also be noted that the *Il1b, Il8* and *Tnfa* gene transcripts have been reported to be increased in traumatic impact injuries in postmortem tissues from human brains [91].

In the mouse, inflammation gene transcripts that increased in abundance included: mitogen-activated protein kinase (*Map3k2*), TNF receptors (*Tnfrsf9, Tnfrs14*), B-cell lymphoma 6 protein (*Bcl6*), C-C chemokine receptor-type 4 (*Ccr4*), Prokineticin-2 (*Prok2*) and platelet-activating factor receptor (*Pafr*) (figure 5). The *Map3k2* gene encodes a kinase that activates pro-inflammatory NF-κB genes [89]. The *Tnfrsf9* and *Tnfrs14* genes encode receptor proteins that have pro-inflammatory functions [83]. The *Bcl6* gene encodes a transcription factor that has anti-inflammatory functions [92]. The *Ccr4* gene encodes a cytokine receptor protein associated with inflammation [74]. The *Prok2 gene* encodes a cytokine-like molecule, while the *Pafr* gene encodes a lipid mediator; both have pro-inflammatory functions [93,94].

Most inflammation-associated gene transcripts increased in abundance within 1 h postmortem and continued to be abundant for 12–48 h. The anti-inflammatory *Bcl6* gene transcripts increased in abundance at two different times, 0.5–6 h and 24 h, suggesting that their abundances might be regulated by a feedback loop. It should also be noted that pro-inflammatory *Map3k2* and *Tnfrs14* gene transcripts were not at high abundance after 24 and 12 h, respectively, which also suggests regulation by a putative feedback loop from the *Bcl6* transcript product.

#### 3.4.1. Summary of inflammation response

In both organisms, some transcripts that increased in abundance have pro-inflammatory functions while others have anti-inflammatory functions. It is possible that the increases in transcript abundances are regulated by feedback loops involving an initial inflammatory reaction followed by an anti-inflammatory reaction to repress it [95]. The variation in the gene transcript abundances of these inflammatory genes suggests the underlying regulatory network is still active in organismal death.

### 3.5. Apoptosis and related genes

Since apoptotic processes kill damaged cells for the benefit of the organism as a whole, we anticipated a significant increase in the abundance of apoptosis gene transcripts in organismal death.

**Figure 6.**
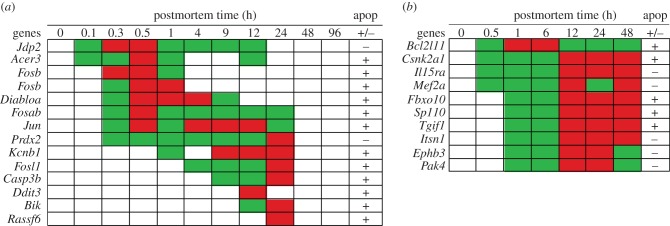
Abundance of apoptosis gene transcripts by postmortem time (h): (*a*) zebrafish and (*b*) mouse. Apoptosis, pro-, +; anti, −. Green, intermediate value; red, maximum value. The *Fosb* gene was represented by two different probes.

In the zebrafish, apoptosis gene transcripts that increased in abundance included: Jun (*Jdp2, Jun*), Alkaline ceramidase 3 (*Acer3*)*, Fos* (*Fosb, Fosab, Fosl1*), IAP-binding mitochondrial protein A (*Diabloa*), Peroxiredoxin-2 (*Prdx2*), Potassium voltage-gated channel member 1 (*Kcnb1*), Caspase apoptosis-related cysteine peptidase 3b (*Casp3b*), DNA-damage-inducible transcript 3 (*Ddit3*), BCL2 (B-cell lymphomas 2)-interacting killer (*Bik*) and Ras association domain family 6 (*Rassf6*) (figure 6). The *Jdp2* gene encodes a protein that represses the activity of the transcription factor activator protein 1 (*AP-1*) [96]. The *Acer3* gene encodes an enzyme that maintains cell membrane integrity/function and promotes apoptosis [97]. The *Fos* genes encode proteins that dimerize with *Jun* proteins to form part of the *AP-1* that promotes apoptosis [98,99]. The *Diabloa* gene encodes a protein that neutralizes inhibitors of apoptosis (IAP)-binding protein [99] and activates caspases [100]. The *Prdx2* gene encodes antioxidant enzymes that control cytokine-induced peroxide levels and inhibit apoptosis [101]. Although the *Kcnb1* gene encodes a protein used to make ion channels, the accumulation of these proteins in the membrane promotes apoptosis via a cell signalling pathway [102]. The *Casp3b* encodes a protein that plays a role in the execution phase of apoptosis [103]. The *Ddit3* gene encodes a transcription factor that promotes apoptosis. The *Bik* gene encodes a protein that promotes apoptosis [104]. The *Rassf6* gene encodes a protein that promotes apoptosis [105].

In the zebrafish, transcripts of both anti-apoptosis *Jdp2* and pro-apopotosis *Acer3* genes increased in abundances within 0.1 h postmortem (figure 6). These increases were followed by increases of five pro-apoptosis gene transcripts and one anti-apoptosis gene transcript within 0.3–0.5 h. The transcriptional dynamics varied among the genes. Specifically, (i) the increased abundance of the *Fosb* gene transcript stopped after 1 h, (ii) the transcripts of the *Diabloa* and *Fosab* genes reached abundance maxima at 0.5–4 h and then their abundances decreased after 9 h for the *Diabloa* and after 24 h for the *Fosab* genes, (iii) the *Jun* gene transcripts reached two maxima (one at 0.5 and another at 4–12 h)—then its abundance decreased after 24 h, and (iv) the transcript of the *Prdx2* gene showed a continuous increase in abundance until reaching a maximum at 24 h and then the abundance decreased. The remaining genes were pro-apoptosis and their transcripts increased in abundance after 1–24 h postmortem. The transcripts of the *Ddit3* and *Rassf6* genes were very different from the other transcripts because they increased in abundance at one sampling time (12 h and 24 h, respectively) and then decreased. Apparently none of the transcripts of apoptosis genes increased in abundance after 24 h, in contrast to genes in other categories (e.g. transcripts of some of stress and immunity genes increased in abundance up to 96 h postmortem).

In the mouse, apoptosis gene transcripts that increased in abundance included: BCL2-like protein 11 (*Bcl2L11*), Casein kinase IIa (*Csnk2a1*), Interleukin 15 receptor subunit a (*Il15ra*), Myocyte enhancer factor 2 (Mef2a), F-box only protein 10 (*Fbxo10*), Sp110 nuclear body protein (*Sp110*), TGFB-induced factor homeobox 1 (*Tgif1*), Intersectin 1 (*Itsm1*), the Ephrin type-B receptor 3 (*Ephb3*) and the p21 protein-activated kinase 4 (*Pak4*) (figure 6). The *Bcl2L11* gene encodes a protein that promotes apoptosis [106]. The *Csnk2a1* gene encodes an enzyme that phosphorylates substrates and promotes apoptosis [107]. The *Il15ra* gene encodes an anti-apoptotic protein [108]. The *Mef2a* gene encodes a transcription factor that prevents apoptosis [109]. The *Fbxo10* gene encodes a protein that promotes apoptosis [110]. The *Sp110* gene encodes a regulator protein that promotes apoptosis [111]. The *Tgif1* gene encodes a transcription factor that blocks signals of the transforming growth factor beta (*TGFβ*) pathway, and therefore is pro-apoptosis [112]. The *Itsn1* gene encodes an adaptor protein that is anti-apoptosis [113]. The *Ephb3* gene encodes a protein that binds ligands on adjacent cells for cell signalling and suppresses apoptosis [108]. The *Pak4* gene encodes a protein that delays the onset of apoptosis [114].

In the mouse, transcripts for the pro- and anti-apoptosis genes increased in abundance within 0.5 h postmortem; however, with the exception of *Bcl2L11*, most reached transcript abundance maxima at 12–48 h postmortem (figure 6). The *Bcl2L11* transcripts reached abundance maxima at 1 and 6 h postmortem.

#### 3.5.1. Summary of apoptotic response

In both organisms, transcripts of both pro- and anti-apoptosis genes increased in abundance in organismal death. However, the timings of the increases, the transcript maximum abundance and the duration of the increased abundances varied by organism. The results suggest the apoptotic genes and their regulation are distinctly different in the zebrafish than the mouse, with transcripts of the mouse genes having increased abundance to 48 h postmortem while those of the zebrafish having increased abundance to 24 h. Nonetheless, the pro- and anti-apoptosis genes appear to be inter-regulating each another.

### 3.6. Transport gene response

Transport processes maintain ion/solute/protein homeostasis and are involved in influx/efflux of carbohydrates, proteins, signalling molecules and nucleic acids across membranes. Transcripts of transport genes should increase in abundance in organismal death in response to dysbiosis.

**Figure 7.**
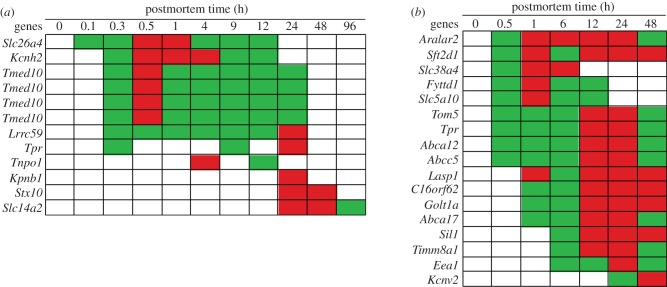
Abundance of transport gene transcripts by postmortem time (h): (*a*) zebrafish and (*b*) mouse. Green, intermediate value; red, maximum value. The *Tmed10* gene was represented by four different probes.

In the zebrafish, transport-associated gene transcripts that increased in abundance included: Solute carrier family 26 anion exchanger member 4 (*Slc26a4*), Potassium channel voltage-gated subfamily H (*Kcnh2*), Transmembrane emp24 domain-containing protein 10 (*Tmed10*), Leucine-rich repeat-containing 59 (*Lrrc59*), the Nucleoprotein TPR (*Tpr*), Importin subunit beta-1 (*Kpnb1*), Transportin 1 (*Tnpo1*), Syntaxin 10 (*Stx10*) and Urea transporter 2 (*Slc14a2*) (figure 7). Of note, the four *Tmed10* transcripts shown in figure 7 each represents a profile targeted by an independent probe. The transcription profiles of this gene were identical indicating high reproducibility of the Gene Meter approach. The *Slc26a4* gene encodes prendrin that transports negatively charged ions (i.e. Cl^−^, bicarbonate) across cellular membranes [115]. The *Kcnh2* gene encodes a protein used to make potassium channels and is involved in signalling [116]. The *Tmed10* gene encodes a membrane protein involved in vesicular protein trafficking [117]. The *Lrrc59*, *Tpr, Tnpo1* and *Kpnb1* genes encode proteins involved in trafficking across nuclear pores [118–121]. The *Stx10* gene encodes a protein that facilitates vesicle fusion and intracellular trafficking of proteins to other cellular components [122]. The *Slc14a2* gene encodes a protein that transports urea out of the cell [123].

The transcripts of *Slc26a4, Kcnh2, Lrrc59* and *Tpr* genes initially increased in abundance within 0.3 h postmortem and remained in high abundance for 12–24 h. The transcripts of the *Tnpo1* gene increased in abundance twice, at 4 and 12 h, suggesting putative regulation by a feedback loop. The transcripts of the remaining genes increased in abundance at 24 h. The increased abundance of the *Slc14a2* gene transcript suggests a build up of urea in zebrafish cells at 24–96 h postmortem, which could be due to the accumulation of urea under hypoxic conditions by the *Arg2* gene (see *Hsp* stress response section).

In the mouse, transport-associated gene transcripts that increased in abundance included: Calcium-binding mitochondrial carrier protein (*Aralar2*), Sodium-coupled neutral amino acid transporter 4 (*Slc38a4*), SFT2 domain-containing 1 (*Sft2d1*), Uap56-interacting factor (*Fyttd1*), Solute carrier family 5 (sodium/glucose co-transporter) member 10 (*Slc5a10*), Mitochondrial import receptor subunit (*Tom5*), Translocated promoter region (*Tpr*), ATP-binding cassette transporter 12 (*Abca12*), Multidrug resistant protein 5 (*Abc5*), LIM and SH3 domain-containing protein (*Lasp1*), Chromosome 16 open reading frame 62 (*C16orf62*), Golgi transport 1 homologue A (*Golt1a*), ATP-binding cassette transporter 17 (*Abca17*), Nucleotide exchange factor (*Sil1*), Translocase of inner mitochondrial membrane 8A1 (*Timm8a1*), Early endosome antigen 1 (*Eea1*) and Potassium voltage-gated channel subfamily V member2 (*Kcnv2*) (figure 7). The *Aralar2* gene encodes a protein that catalyses calcium-dependent exchange of cytoplasmic glutamate with mitochondrial aspartate across the mitochondrial membrane and may function in the urea cycle [124]. The *Slc38a4* gene encodes a symport that mediates transport of neutral amino acids and sodium ions [125]. The *Sft2d1* gene encodes a protein involved in transporting vesicles from the endocytic compartment of the Golgi complex [126]. The *Fyttd1* gene is responsible for mRNA export from the nucleus to the cytoplasm [127]. The *Slc5a10* gene encodes a protein that catalyses carbohydrate transport across cellular membranes [128]. The *Tom5* gene encodes a protein that plays a role in importation to proteins destined for mitochondrial sub-compartments [129]. The *Abca12*, *Abca17* and *Abc5* genes encode proteins that transport molecules across membranes [130–132]. The *Lasp1* gene encodes a protein that regulates ion transport [133]. The *C16orf62* gene encodes a protein involved in protein transport from the Golgi apparatus to the cytoplasm [134]. The *Golt1a* gene encodes a vesicle transport protein [126]. The *Sil1* gene encodes a protein involved in protein translocation into the endoplasmic reticulum [135]. The *Timm8a1* gene encodes a protein that assists importation of other proteins across inner mitochondrial membranes [136]. The *Eea1* gene encodes a protein that acts as a tethering molecule for vesicular transport from the plasma membrane to the early endosomes [137]. The *Kcnv2* gene encodes a membrane protein involved in generating action potentials [138].

Within 0.5 h postmortem, transcripts of genes involved in: (i) ion and urea regulation (*Aralar*), (ii) amino acid (*Slc38a4*), carbohydrate (*Slc5a10*) and protein (*Sft2d1, Tom5*) transport, (iii) mRNA nuclear export (*Fyttd1, Tpr*) and (iv) molecular efflux (*Abca12, Abc5*) increased in abundance in the mouse. The transcription profiles of these genes varied in terms of transcript abundance maxima and duration. While the transcripts of *Aralar, Sft2d1, Slc38a4, Fyttd1* and *Slc5a10* reached abundance maxima at 1 h, those of *Tom5, Tpr, Abca12* and *Abc5* reached maxima at 12–24 h postmortem. The duration of the increased abundance also varied for these transcripts since most remained at high abundances for 48 h postmortem, while the *Sft2d1, Fyttd1* and *Slc5a10* transcripts were at high abundances from 0.5 to 12+ h. The shorter duration of increased abundance suggests prompt gene repression. The transcript abundances of *Lasp1, C16orf62, Golt1a* and *Abca17* increased at 1 h postmortem and remained elevated for 48 h. The transcripts of *Sil1, Timm8a1* and *Eea1* increased in abundance at 6 h, while those of *Kcnv2* increased at 24 h postmortem and remained elevated for 48 h.

#### 3.6.1. Summary of transport genes

The increased abundance of transcripts of transport genes suggests attempts by zebrafish and mice to reestablish homeostasis. Although the transcripts of half of these genes increased in abundance within 0.5 h postmortem, many increased at different times and for varying durations. While most of the transcripts of transport genes in the zebrafish were not abundant after 24 h, most transcripts of transport genes in the mouse remained abundant for 24–48 h postmortem.

### 3.7. Developmental control genes

An unexpected finding in this study was the increased abundance of transcripts of developmental control genes in organismal death. Developmental control genes are mostly involved in regulating developmental processes from early embryo to adult in the zebrafish and mouse; therefore, we did not anticipate their transcripts to become more abundant in organismal death.

**Figure 8.**
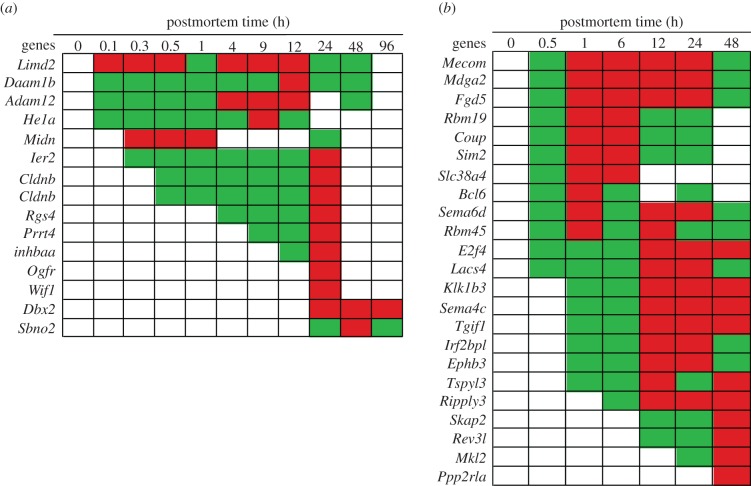
Abundance of development gene transcripts by postmortem time (h): (*a*) zebrafish and (*b*) mouse. Green, intermediate value; red, maximum value. The *Cldnb* gene was represented by two different probes.

In the zebrafish, development-associated gene transcripts that increased in abundance included: LIM domain-containing protein 2 (*Limd2*)*,* Disheveled-associated activator of morphogenesis 1 (*Daam1b*)*,* Meltrin alpha (*Adam12*)*,* Hatching enzyme 1a (*He1a*)*,* Midnolin (*Midn*)*,* Immediate early response 2 (*Ier2*)*,* Claudin b (*Cldnb*)*,* Regulator of G-protein signalling 4-like (*Rgs4*)*,* Proline-rich transmembrane protein 4 (*Prrt4*)*,* Inhibin (*Inhbaa*)*,* Wnt inhibitory factor 1 precursor (*Wif1*), Opioid growth factor receptor (*Ogfr*)*,* Strawberry notch homolog 2 (*Sbno2*) and Developing brain homeobox 2 (*Dbx2*) (figure 8). The *Limd2* gene encodes a binding protein that plays a role in zebrafish embryogenesis [139]. The *Daam1b* gene regulates endocytosis during notochord development [140]. The *Adam12* gene encodes a metalloprotease-disintegrin involved in myogenesis [141]. The *He1a* gene encodes a protein involved in egg envelope digestion [142]. The *Midn* gene encodes a nucleolar protein expressed in the brain that is involved in the regulation of neurogenesis [143,144]. The *Ier2* gene encodes a protein involved in left–right asymmetry patterning in the zebrafish embryo [145]. The *Cldnb* gene encodes a tight junction protein in larval zebrafish [146]. The *Rgs4* gene encodes a protein involved in brain development [147]. The *Prrt4* gene encodes a protein that is predominantly expressed in the brain and spinal cord in embryonic and postnatal stages of development. The *Inhbaa* gene encodes a protein that plays a role in oocyte maturation [148]. The *Wif1* gene encodes a WNT inhibitory factor that controls embryonic development [149]. The *Ogfr* gene plays a role in embryonic development [150]. The *Sbno2* gene plays a role in zebrafish embryogenesis [151]. The *Dbx2* gene encodes a transcription factor that plays role in spinal cord development [152].

Although the abundances of *Limd2, Daam1, Adam12 and He1a* transcripts increased in the zebrafish within 0.1 h postmortem, other gene transcripts in this category increased from 0.3 to 24 h postmortem, reaching abundance maxima at 24 h or more.

In the mouse, development-associated gene transcripts that increased in abundance included: MDS1 and EVI1 complex locus protein EVI1 (*Mecom*)*,* MAM domain-containing glycosylphosphatidylinositol anchor 2 (*Mdga2*)*,* FYVE, RhoGEF and PH domain-containing 5 (*Fgd5*)*,* RNA-binding motif protein 19 (*Rbm19*)*,* Chicken ovalbumin upstream promoter (*Coup*)*,* Single-minded homolog 2 (*Sim2*)*,* Solute carrier family 38, member 4 (*Slc38a4*)*,* B-cell lymphoma 6 protein (*Bcl6*)*,* Sema domain transmembrane domain (TM) cytoplasmic domain (semaphorin) 6D (*Sema6d*)*,* RNA binding motif protein 45 (*Rbm45*)*,* Transcription factor E2F4 (*E2f4*)*,* Long chain fatty acid-CoA ligase 4 (*Lacs4*)*,* Kallikrein 1-related peptidase b3 (*Klk1b3*)*,* Sema domain, immunoglobulin domain, TM and short cytoplasmic domain (*Sema4c*)*,* TGFB-induced factor homeobox 1 (*Tgif1*)*,* Interferon regulatory factor 2-binding protein-like (*Irf2bpl*)*,* Ephrin type-B receptor 3 (*Ephb3*)*,* Testis-specific Y-encoded-like protein 3 (*Tspyl3*)*,* Protein ripply 3 (*Ripply3*)*,* Src kinase-associated phosphoprotein 2 (Skap2)*,* DNA polymerase zeta catalytic subunit (*Rev3l*)*,* MKL/myocardin-like 2 (*Mkl2*) and Protein phosphatase 2 regulatory subunit A (*Ppp2r1a*) (figure 8). The *Mecom* gene plays a role in embryogenesis and development [153]. The *Mdga2* gene encodes immunoglobins involved in neural development [154]. The *Fgd5* gene is needed for embryonic development since it interacts with hematopoietic stem cells [155]. The *Rbm19* gene is essential for preimplantation development [156]. The *Coup* gene encodes a transcription factor that regulates development of the eye [157] and other tissues [158]. The *Sim2* gene encodes a transcription factor that regulates cell fate during midline development [159]. The *Slc38a4* gene encodes a regulator of protein synthesis during liver development and plays a crucial role in fetal growth and development [160,161]. The *Bcl6* gene encodes a transcription factor that controls neurogenesis [162]. The *Sema6d* gene encodes a protein involved in retinal development [163]. The *Rbm45* gene encodes a protein that has preferential binding to poly(C) RNA and is expressed during brain development [164]. The *E2f4* gene is involved in maturation of cells in tissues [165]. The *Lacs4* gene plays a role in patterning in embryos [166]. The *Klk1b3* gene encodes a protein that plays a role in the developing embryos [167]. The *Sema4c* gene encodes a protein that has diverse function in neuronal development and heart morphogenesis [168,169]. The *Tgif1* gene encodes a transcription factor that plays a role in trophoblast differentiation [170]. The *Irf2bpl* gene encodes a transcriptional regulator that plays a role in female neuroendocrine reproduction [171]. The *Ephb3* gene encodes a kinase that plays a role in neural development [172]. The *Tspyl3* gene plays a role in testis development [173]. The *Ripply3* gene encodes a transcription factor involved in development of the ectoderm [174]. The *Skap2* gene encodes a protein involved in actin reorganization in lens development [175]. The *Rev3l* gene encodes a polymerase that can replicate past certain types of DNA lesions and is necessary for embryonic development [176]. The *Mkl2* gene encodes a transcriptional co-activator that is involved in the formation of muscular tissue during embryonic development [177]. The *Ppp2r1a* gene plays a role in embryonic epidermal development [178].

The transcripts of *Mecom, Mdga2, Fgd5, Rbm19, Coup, Sim2, Slc38a4, Bcl6, Sema6d, Rbm45, E2f4* and *Lacs4* genes in the mouse significantly increased in abundance within 0.5 h postmortem but the other transcripts increased from 1 h to 48 h reaching abundance maxima at 12 h or more.

#### 3.7.1. Summary of developmental control genes

In organismal death, there is progressive increase in transcript abundances of some developmental control genes suggesting that they are no longer silenced. A possible reason for these increased abundances is that the postmortem physiological conditions resemble those of earlier developmental stages.

### 3.8. Cancer genes

There are a number of databases devoted to cancer and cancer-related genes. Upon cross-referencing the genes found in this study, we discovered a significant overlap. The genes found in this search are presented below.

**Figure 9.**
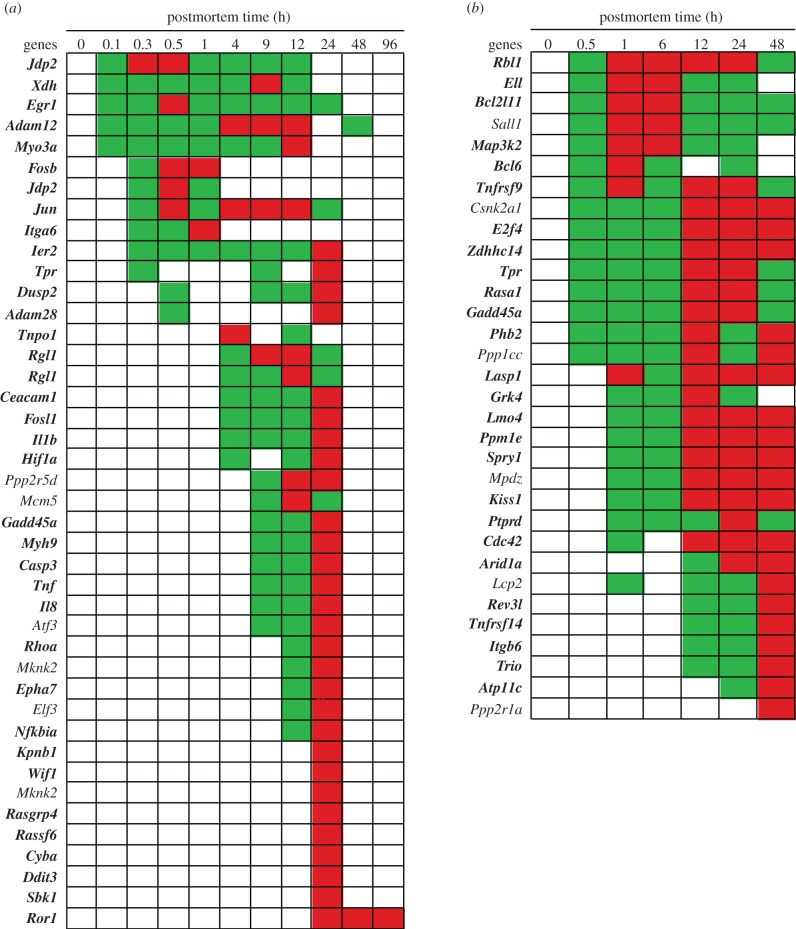
Abundance of cancer gene transcripts by postmortem time (h): (*a*) zebrafish and (*b*) mouse. Green, intermediate value; red, maximum value. Bold gene name means it was found in more than one cancer database. The *Rgl1* gene was represented by two different probes.

In the zebrafish, transcripts of the following cancer genes significantly increased in abundance: *Jdp2*, Xanthine dehydrogenase (*Xdh*), *Egr1, Adam12*, Myosin-IIIa (*Myo3a*), *Fosb, Jun,* Integrin alpha 6b (*Itga6*), *Ier2, Tpr,* Dual specificity protein phosphatase 2 (*Dusp2*), Disintegrin and metallopeptidase domain 28 (*Adam28*), *Tnpo1,* Ral guanine nucleotide dissociation stimulator-like (*Rgl1*), Carcinoembryonic antigen-related cell adhesion molecule 5 (*Ceacam1*), *Fosl1, Il1b, Hif1a,* Serine/threonine-protein phosphatase 2A regulatory (*Ppp2r5d*), DNA replication licensing factor (*Mcm5*), *Gadd45,* Myosin-9 (*Myh9*), *Casp3, Tnf, Il8,* Cyclic AMP-dependent transcription factor (*Atf3*), small GTPase (*RhoA*), *Mknk2,* Ephrin type-A receptor 7 precursor (*Epha7*), ETS-related transcription factor (*Elf3*), *Nfkbia, Kpnb1, Wif1,* RAS guanyl-releasing protein 1 (*Rasgrp*), Ras association domain-containing protein 6 (*Rassf6*), *Cyba,* DNA-damage-inducible transcript 3 (*Ddit3*), Serine/threonine-protein kinase (*Sbk1*) and Tyrosine-protein kinase transmembrane receptor (*Ror1*) (figure 9).

In the mouse, transcripts of the following cancer genes significantly increased in abundance: Retinoblastoma-like protein 1 (*Rbl1*), Elongation factor RNA polymerase II (*Ell*), Bcl-2-like protein 11 (*Bcl2l11*), Sal-like protein 1 (*Sall1*), *Map3k2, Bcl6, Tnfrsf9,* CK2 target protein 2 (*Csnk2a1*), Transcription factor E2f4 (*E2f4*), Zinc finger DHHC-type containing 14 (*Zdhhc14*), *Tpr,* RAS p21 protein activator 1 (*Rasa1*), *Gadd45,* Prohibitin (*Phb2*), Serine/threonine-protein phosphatase PP1-gamma catalytic (*Ppp1cc*), *Lasp1,* G protein-coupled receptor kinase 4 (*Grk4*), LIM domain transcription factor (*Lmo4*), Protein phosphatase 1E (*Ppm1e*), Protein sprouty homolog 1 (*Spry1*), Multiple PDZ domain protein (*Mpdz*), Kisspeptin receptor (*Kiss1*), Receptor-type tyrosine-protein phosphatase delta precursor (*Ptprd*), Small effector protein 2-like (*Cdc42*), AT-rich interactive domain-containing protein 1A (*Arid1a*), Lymphocyte cytosolic protein 2 (*Lcp2*), DNA polymerase zeta catalytic subunit (*Rev3l*), *Tnfrsf14,* Integrin beta-6 precursor (*Itgb6*), Triple functional domain protein (*Trio*), ATPase class VI type 11C (*Atp11c*) and Serine/threonine-protein phosphatase 2A regulatory (*Ppp2r1a*) (figure 9).

#### 3.8.1. Summary of cancer genes

Genes analysed under this category were classified as ‘cancer genes’ in a Cancer Gene Database [10] (figure 9). The timing, duration and peak transcript abundances differed within and between organisms. Note that some gene transcripts had two abundance maxima. In the zebrafish, this phenomenon occurred for *Adam12, Jun, Tpr, Dusp2, Tnpo1* and *Hif1a* genes and in the mouse, *Bcl6, Tnfrs9, Lasp1, Cdc42* and *Lcp2* genes, and is consistent with the notion that the transcript abundances are being regulated through feedback loops.

### 3.9. Epigenetic regulatory genes

Epigenetic regulation of gene expression involves DNA methylation and histone modifications of chromatin into active and silenced states [179]. These modifications alter the condensation of the chromatin and affect the accessibility of the DNA to the transcriptional machinery. Although epigenetic regulation plays an important role in development, modifications can arise stochastically with age or in response to environmental stimuli [180]. Hence, we anticipated that epigenetic regulatory genes would be involved in organismal death.

**Figure 10.**
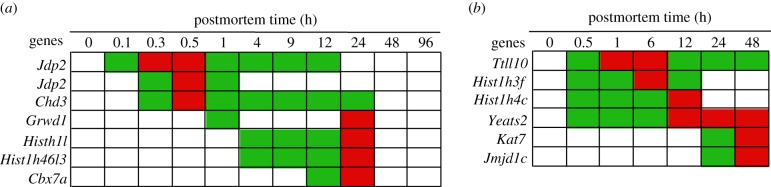
Abundance of epigenetic gene transcripts by postmortem time (h): (*a*) zebrafish and (*b*) mouse. Green, intermediate value; red, maximum value. Bold gene name means it was found in more than one cancer database. The *Jdp2* gene was represented by two different probes.

In the zebrafish, transcripts of the following epigenetic genes significantly increased in abundance: Jun dimerization protein 2 (*Jdp2*), Chromatin helicase protein 3 (*Chd3*), Glutamate-rich WD repeat-containing protein 1 (*Grwd1*), Histone H1 (*Histh1l*), Histone cluster 1, H4-like (*Hist1h46l3*) and Chromobox homolog 7a (*Cbx7a*) (figure 10). The *Jdp2* gene is thought to inhibit the acetylation of histones and repress expression of the *c-Jun* gene [181]. The *Chd3* gene encodes a component of a histone deacetylase complex that participates in the remodelling of chromatin [182]. The *Grwd1* gene is thought to be a histone-binding protein that regulates chromatin dynamics at the replication origin [183]. The *Histh1l* gene encodes a histone protein that binds the nucleosome at the entry and exit sites of the DNA and the *Hist1h46l3* gene encodes a histone protein that is part of the nucleosome core [184]. The *Cbx7a* gene encodes an epigenetic regulator protein that binds non-coding RNA and histones and represses gene expression of a tumor suppressor [185].

The transcripts of both *Jdp2* and *Chd3* genes increased in abundance within 0.3 h postmortem, and reached abundance maxima at 0.5 h. Note that two different probes targeted the *Jdp2* transcript. The transcript of the *Grwd1* gene increased in abundance at 1 h and 24 h postmortem. The transcript of the histone genes increased in abundance at 4 h postmortem, reaching abundance maxima at 24 h. The transcript of the *Cbx7a* gene increased in abundance at 12 h, reaching an abundance maximum at 24 h. The transcript abundances of these genes decreased after 24 h.

In the mouse, transcripts of the following epigenetic genes significantly increased in abundance: Tubulin tyrosine ligase-like family member 10 (*Ttll10*), Histone cluster 1 H3f (*Hist1h3f*), Histone cluster 1 H4c (*Hist1h4c*), YEATS domain-containing 2 (*Yeats2*), Histone acetyltransferase (*Kat7*) and Probable JmjC domain-containing histone demethylation protein 2C (*Jmjd1c*) (figure 10). The *Ttll10* gene encodes a polyglycylase involved in modifying nucleosome assembly protein 1 that affects transcriptional activity, histone replacement and chromatin remodelling [186]. The *Hist1h3f* and *Hist1h4c* genes encode histone proteins that are the core of the nucleosomes [187]. The *Yeats2* gene encodes a protein that recognizes histone acetylations so that it can regulate gene expression in the chromatin [188]. The *Kat7* gene encodes an acetyltransferase that is a component of histone-binding origin-of-replication complex, which acetylates chromatin and therefore regulates DNA replication and gene expression [189]. The *Jmjd1c* gene encodes an enzyme that specifically demethylates Lys-9 of histone H3 and is implicated in the reactivation of silenced genes [190].

The transcripts of the *Ttll10, Yeats2* and histone protein genes increased in abundance 0.5 h postmortem and reached maxima at different times, with the *Ttll10* transcript reaching a maximum at 1 to 6 h, the histone transcripts reaching maxima at 6 and 12 h postmortem, and the *Yeats2* transcript reaching maxima at 12–24 h postmortem (figure 10). The transcripts of the *Kat7* and *Jmjd1c* genes increased in abundance at 24 h, reaching abundance maxima at 48 h postmortem. Note that the transcripts of the histone genes were no longer abundant after 24 h postmortem.

#### 3.9.1. Summary of epigenetic regulatory genes

The increased abundance of transcripts of genes encoding histone proteins, histone–chromatin modifying proteins, and proteins involved in regulating DNA replication at the origin were common to the zebrafish and the mouse. These findings indicate that epigenetic regulatory genes are still modifying chromatin structure in organismal death and thus change the accessibility of transcription factors to the promoter or enhancer regions.

### 3.10. Percentage of gene transcripts with significant abundance by postmortem time

**Figure 11.**
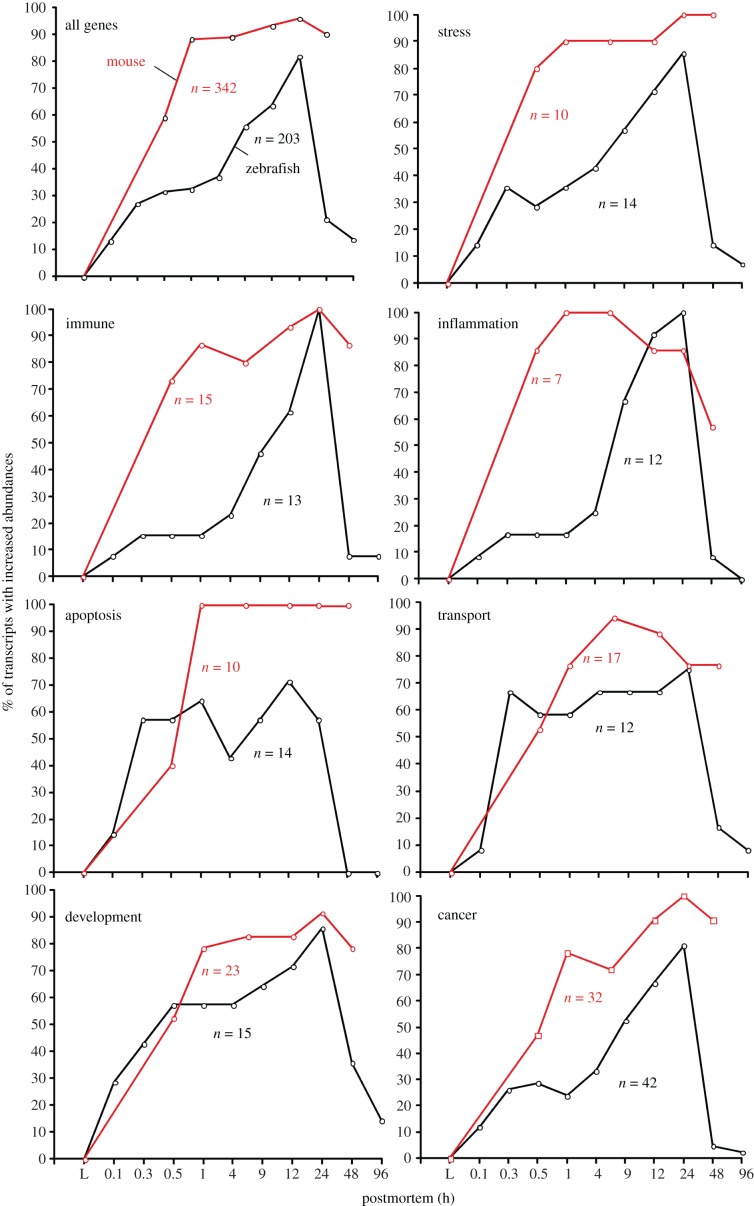
Percentage of transcripts with increased abundances by postmortem time and category. Number of total genes by organism and category are shown. ‘All genes’ refer to the gene transcripts that significantly contributed to the ordination plots. Mouse is red and zebrafish is black.

The percentage of gene transcripts was defined as the number of gene transcripts with abundances greater than the control over the total number of transcripts with significant abundance in a category at a specific postmortem time. A comparison of the percentage of gene transcripts by postmortem time of all gene categories revealed similarities between the zebrafish and the mouse. Specifically, most gene transcripts increased in abundance between 0.5 and 24 h postmortem, and after 24 h the transcript abundance drastically dropped (figure 11, ‘All genes’). It should be noted that the same pattern was found in stress, transport and development categories for both organisms. However, in the zebrafish, the immunity, inflammation, apoptosis and cancer categories differed from the mouse. Specifically, the gene transcripts in the immunity, inflammation and cancer categories increased in abundance much later (1–4 h) in the zebrafish than the mouse, and the duration of elevated abundances was much shorter. For example, while 90% of the transcripts for genes in the immunity and inflammation categories increased in abundance in the mouse within 1 h postmortem, less than 30% of the transcripts in the same categories were abundant in the zebrafish (figure 11), indicating a slower initial response. It should be noted that while the number of transcripts of immunity genes reached abundance maxima at 24 h postmortem in both organisms, the number of inflammation genes reaching abundance maxima occurred at 1–4 h in the mouse and 24 h in the zebrafish. The significance of these results is that the inflammation response occurs rapidly and robustly in the mouse while in the zebrafish it takes longer to establish, which could be attributed to phylogenetic differences. There were significant differences in the transcript abundances of apoptosis genes between the zebrafish and the mouse. In the mouse, the percentage of transcripts of apoptosis genes reached 100% at 1 h postmortem and remained sustained for 48 h postmortem, while the percentage of transcript genes with increased abundance in the zebrafish never reached 70% and the abundances abruptly decreased after 12 h.

### 3.11. Upregulation or differential mRNA stability?

Since equal amounts of RNA were used for all time points (see below), although degradation was ongoing, it is theoretically possible that the apparent increase in the abundance of a subset of transcripts is actually due to a higher stability of these transcripts compared to the background of degrading transcripts. Hence, the question arises whether higher transcript abundances are due to upregulation after organismal death or complex decay profiles leading to relative enrichments. To determine whether the significant increases were due to such an enrichment, the expected profile of a hypothetical stable non-degrading cRNA was calculated for the zebrafish, mouse liver and mouse brain. In theory, the abundance of a stable non-degrading cRNA transcript determined by the Gene Meter approach should positively correlate to the amount of total cRNA delivered to the DNA microarray. Below is a rationale and the approach used to identify stable non-degrading cRNAs in the transcript pool.

As outlined in the Material and methods section, the amount of sample taken from an animal was approximately the same and the homogenization volume was the same. The electronic supplementary material, tables S1 and S2 show the quantity of total RNA extracted from a tissue (*x*, ng µl^−1^). Since a fixed amount of RNA was taken into labelling, the volume of the homogenized sample was proportional to 1/*x*, i.e. the effective quantity of tissue taken into the microarray analysis was proportional to 1/*x*.

Let us assume there was a subset of stable RNAs, while all other RNA molecules were degrading. Hence, the quantity of the stable gene transcript would be directly proportional to the amount of tissue taken into the experiment, 1/*x*. In order to provide an expected concentration–time profile for the assumed stable cRNA, one can compare (on the log2 scale) the 1/*x* values for all time points. To make it relative to the live control, the log2 value of the control will be subtracted from each time point. The obtained profile will be the expected profile (fold change) of a stable non-degrading cRNA.

**Figure 12.**
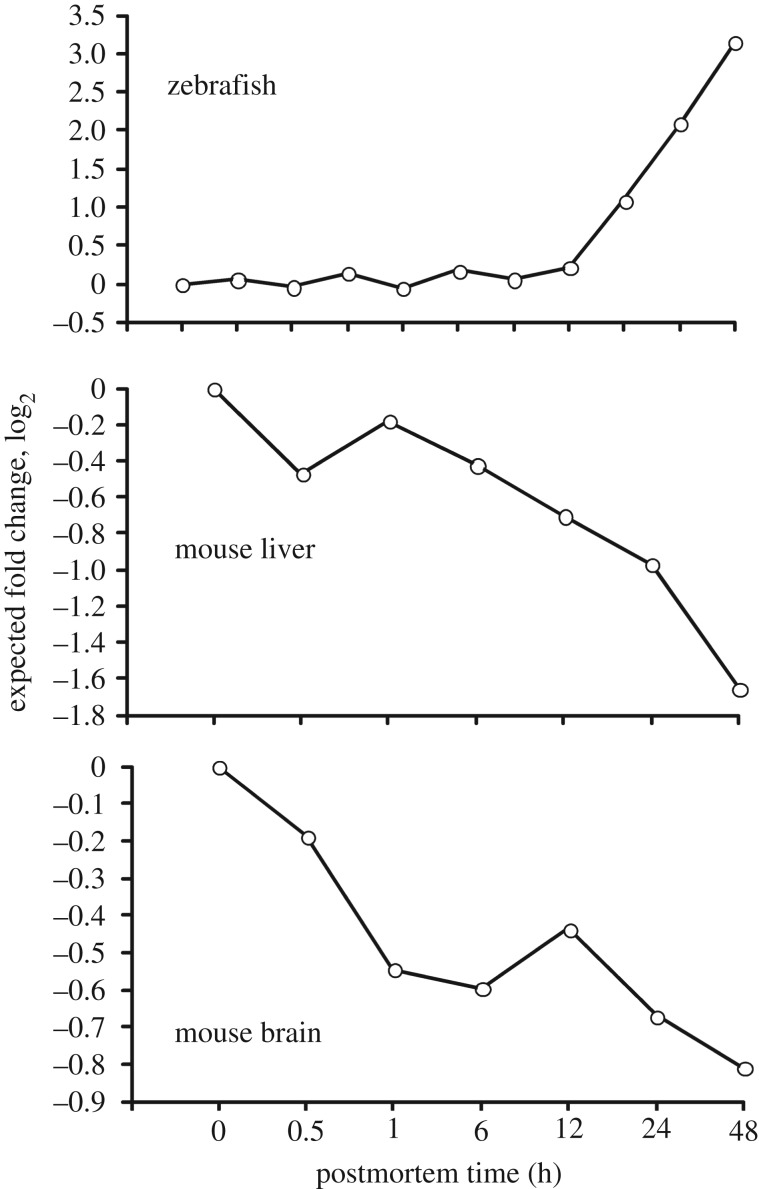
Expected fold change of a putatively stable cRNA by postmortem time. Fold change was determined by subtracting the log2 of the inverted concentration in µl ng^−1^ of the extracted cRNA of the live controls from the inverted concentration of extracted cRNA at each sampling time.

Taking into account the above considerations, we found that the expected profile of the stable zebrafish cRNA would have an eightfold increase at 96 h postmortem (figure 12). By contrast, at 48 h postmortem, we found that the expected profile of the stable cRNA in mouse liver would have an approximately fourfold decrease, while the stable cRNA in the mouse brain would have an approximately twofold decrease, because less tissue was taken into the microarray analysis (since the total RNA yield increased). It is important to note that these expected stable mouse mRNA profiles (lower two panels of figure 12) would not have been selected by the statistical procedure for identifying transcriptional profiles that significantly increased in abundance relative to the live controls.

**Figure 13.**
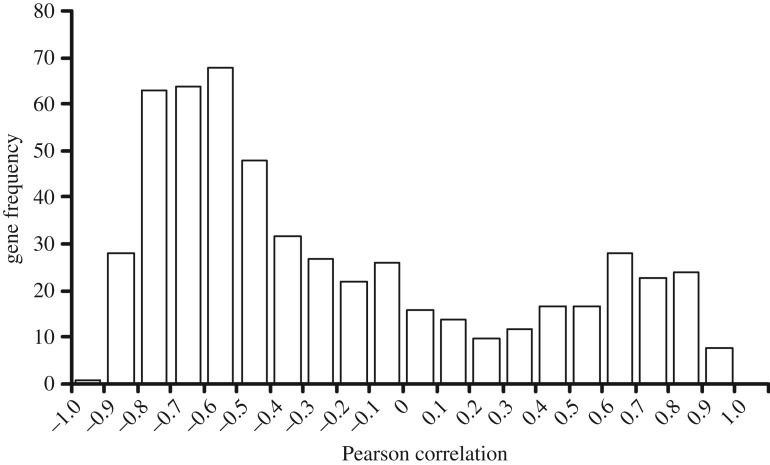
Distribution of the correlations of the expected fold change and relative gene transcript abundance by postmortem time for the zebrafish. We only considered probes targeting gene transcripts that significantly increased with postmortem time relative to the live controls (*n* = 548). Correlations above 0.685 were significant at *α* = 0.01 and indicate the possibility of enrichment of the stable cRNA.

In the zebrafish, the potentially enriched transcripts (due to their stability) were identified by correlating their abundances to the expected fold change of the stable cRNA. In theory, potentially enriched transcripts should be positively correlated to the expected fold change of the putatively stable transcript. Alternatively, transcripts that are not enriched due to stability effects should be negatively correlated or not correlated at all to the expected fold change. The correlations of the 548 gene transcripts (i.e. those that were significantly increased in abundance with postmortem time) ranged from highly negative to highly positive (figure 13). Note higher frequency gene transcripts on the left side of the histogram indicate that most transcripts were negatively correlated to the expected fold change, while the lower frequency on the right side of the histogram indicates a small portion of the gene transcripts were putatively enriched than other transcripts. A standard statistical table, using d.f. = *n* − 2 with direction, revealed that correlations greater than 0.685 were statistically significant at *α* = 0.01 (three bars on the right side of the histogram). Hence, 45 of the 548 gene transcripts were found to be significantly correlated to the expected fold change of the stable cRNA.

**Figure 14.**
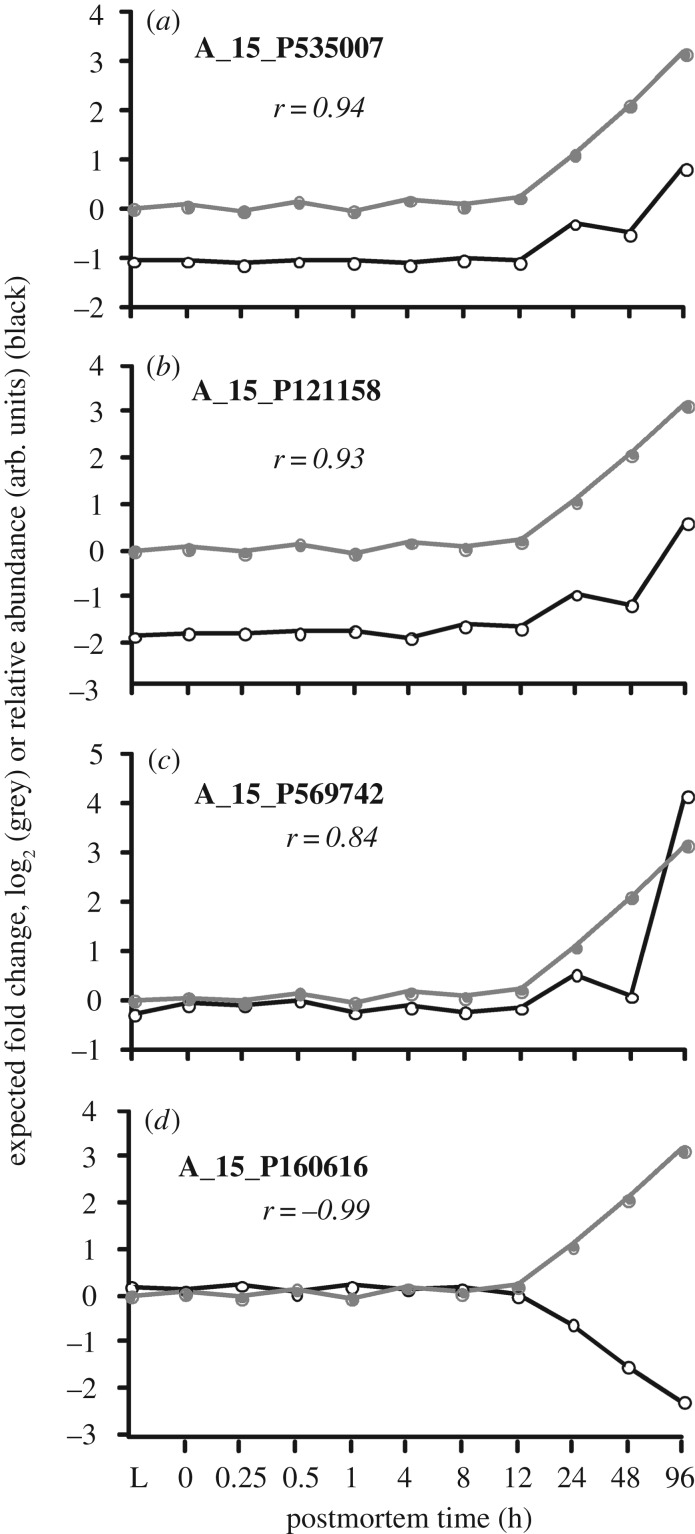
Comparison of expected fold change (grey) based on total cRNA extracted relative to live control versus specific gene transcript profiles (black) by postmortem time. (*a*–*c*) Relative transcript abundances that are highly correlated with expected fold change and are therefore putatively enriched and/or stable. (*d*) A transcript profile that is negatively correlated with expected fold change and therefore neither enriched nor stable.

The 45 gene transcripts that were putatively enriched are shown in table 1. Figure 14 shows the transcriptional profiles of three selected gene transcripts compared to expected fold change profile as shown in the top three panels. The transcriptional profile of a negatively correlated gene transcript served as a control. None of the gene transcripts from the mouse samples were enriched because the amount of cRNA in the tissue extract increased or stayed about the same with postmortem time.

**Table 1.** Positively correlated zebrafish probes and expected fold change at *α* = 0.01. −, non-annotated gene. The profiles of the probes in bold are shown in figure 14.

zebrafish probe	*r*	gene annotation
A_15_P575692	0.69	—
A_15_P573832	0.7	*Arrdc3a*
A_15_P204336	0.71	*Chrnb2*
A_15_P330956	0.71	—
A_15_P100286	0.71	*Tyw5*
A_15_P558612	0.73	—
A_15_P234336	0.73	—
A_15_P499242	0.74	*Il6st*
A_15_P170211	0.79	*Cdh2*
A_15_P262926	0.8	—
A_15_P105618	0.8	*Gpr143*
A_15_P114708	0.88	*Nnmt*
**A_15_P121158**	0.93	*Tf3a*
A_15_P138521	0.9	*Sbno2*
A_15_P147901	0.72	*Slc14a2*
A_15_P154461	0.84	*Dbx2*
A_15_P155766	0.77	*Tox2*
A_15_P157941	0.82	*Minal*
A_15_P165836	0.93	*C3a*
A_15_P188941	0.8	*Rapgef1a*
A_15_P207266	0.78	*Cacnb2a*
A_15_P251041	0.83	—
A_15_P251726	0.72	—
A_15_P254361	0.9	—
A_15_P262011	0.84	—
A_15_P295031	0.84	—
A_15_P309396	0.77	—
A_15_P336700	0.77	*Dhrs7*
A_15_P338120	0.73	—
A_15_P342270	0.82	*Tcf3*
A_15_P348865	0.85	*Prdm16*
A_15_P360015	0.7	*Fhod3*
A_15_P368985	0.86	*Pde4d*
A_15_P388050	0.83	*Spata6l*
A_15_P389300	0.85	*Ror1*
A_15_P407295	0.82	—
A_15_P452885	0.79	*Cfp*
A_15_P470745	0.78	*Nfix*
A_15_P526247	0.83	A*f9*
**A_15_P535007**	0.94	*Psd3*
A_15_P542897	0.74	*Rps6ka3a*
A_15_P544722	0.76	—
A_15_P565217	0.83	—
**A_15_P569742**	0.84	*Efr3a*
A_15_P603052	0.77	*Stard13b*

## 4. Discussion

The primary motivation for our study was driven by curiosity in the processes involved in the shutting down of a complex biological system—which has received little attention so far. Other fields of research have examined the shutdown of complex systems (e.g. societies [191], government [192] and electrical black outs [193]). Yet, to our knowledge, no study has examined long-term postmortem dynamics of transcripts from vertebrates kept in their native conditions. The secondary motivation for our study was to demonstrate the utility of Gene Meter technology for gene expression studies.

### 4.1. Why study transcriptional dynamics in death?

While the development of a complex biological system requires time and energy, its shutdown and subsequent disassembly entails the dissipation of energy and the unravelling of complex structures and could provide novel insights into interesting pathways. Since the shutdown of a complex system does not occur instantaneously, not all cells in a body will be immediately affected by pending death. Hence, the transcript pools should contain mRNAs involved in day-to-day survival as well as stress compensation—yet, since death is the unequivocal end of life—one would expect mRNAs will change with postmortem time. How the transcription pools dynamically change with postmortem time was the goal of this study.

As one would expect, a living system is a collection of biochemical reactions linked together by the components participating in them. These reactions depending on one another to a certain extent, we conjecture that the observed increases in transcript abundance are due to thermodynamic and kinetic conditions that are encountered during organismal death and that reflect, at least in part, pathways that are used during life. For example, the increased abundances of epigenetic regulatory transcripts suggest that histone modification (e.g. *Histh1l*) and chromatin interactions (e.g. *Grwd1, Chd3, Yeats, Jmjd1c*) could be taking place (figure 10). Products of these transcripts could be responsible for the unravelling of the nucleosomes, which enable transcription factors and RNA polymerases to transcribe the developmental control genes previously silenced since embryogenesis (figure 8). Hence, one plausible explanation for the increase in transcript abundances is that specific genes are transcriptionally upregulated. The energy barrier in this example is the tightly wrapped nucleosomes that previously did not allow access to developmental control genes. Other energy or entropy barriers include the nucleopores that allow the exchange of mRNA and other molecules between the mitochondria and the cytosol (e.g. *Tpr, Tnpo1, Lrrc59*), or the ion/solute protein channels (e.g. *Aralar2, Slc38a4*) that control intracellular ions regulating apoptotic pathways [194,195].

### 4.2. Methodological validity

The Gene Meter approach is pertinent to the quality of the microarray output obtained in this study because conventional DNA microarrays yield noisy data [196,197]. The Gene Meter approach determines the behaviour of every microarray probe by calibration—which is analogous to calibrating a pH meter with buffers. Without calibration, the precision and accuracy of a meter is not known, nor can one know how well the experimental data fits to the calibration (i.e. *R^2^*). In the Gene Meter approach, the response of a probe (i.e. its behaviour in a dilution series) is fitted to either a Freundlich or Langmuir adsorption model, and probe-specific parameters are calculated. The ‘noisy’ or ‘insensitive’ probes are identified and removed from further analyses. Probes that sufficiently fit the model are retained and later used to calculate the abundance of a specific gene or gene transcript in a biological sample. The models take into consideration the nonlinearity of the microarray signal and the calibrated probes do not require normalization procedures to compare biological samples. By contrast, conventional DNA microarray approaches are biased because different normalizations can yield up to 20–30% differences in the up- or downregulation depending on the procedure selected [198–201]. Another issue with normalization is that it will artificially increase some of the transcripts due to the denominator. For example, if one normalizes gene abundances to the sum of all transcripts (as often done in conventional microarray studies) and the majority of transcripts degrades with postmortem time, the normalized values of the stable transcripts will be artificially increased due to the decreasing denominator.

We recognize that next-generation sequencing (NGS) approaches could have been used to monitor the increase in transcript abundances in this study. However, the same problems of normalization and reproducibility (mentioned above) are pertinent to NGS technology [202]. Hence, the Gene Meter approach is currently the most advantageous to study postmortem gene transcript abundances in a high-throughput manner. Moreover, a recent publication by a group from the US National Institute of Standards and Technology used the same dilution series approach (as we did in this study) to evaluate and calibrate RNASeq [203]. They found RNASeq comparable to microarrays in terms of target quantification, but not superior, as may be perceived by the community.

### 4.3. Transcription versus decay

Other plausible explanations for the observed increase in transcript abundances include enrichment of stable non-degrading RNA in the transcription pool [204] and/or the changing of cell types in the samples with postmortem time. In this study, we specifically addressed the enrichment issue by identifying 45 gene transcripts in the zebrafish that could have been artificially ‘enriched’ after 12 h postmortem (table 1 and figure 14). In contrast to the zebrafish, we found none of the gene transcripts were enriched in the mouse. The significance of this finding is that a very low percentage (less than 4%) of the 1063 transcriptional profiles examined in our study could be classified as ‘artificially’ enriched due to differential stability. This inference is based on assuming constant decay rates, but formally we cannot exclude the possibility that complex differential stability effects might also occur, which by themselves might be parts of regulatory loops. Hence, it will be of interest in future experiments to assess with more direct means the fraction of genes that get actively transcribed after death versus those that show complex decay patterns.

It should be noted that a small number of genes has been previously reported to be upregulated in cadavers. Using reverse transcription real-time quantitative PCR (RT-RTqPCR), a study showed significant increases in expression of Myosin light chain 3 (*Myl3*), Matrix metalloprotease 9 (*Mmp9*), and Vascular endothelial growth factor A (*Vegfa*) genes in body fluids after 12 h postmortem [205]. Interestingly, we found an increased abundance of myosin-related and matrix metalloprotease transcripts in our study. Specifically, the myosin-related genes included: Myosin-Ig (*Myo1g)* in the mouse*,* and Myosin-IIIa (*Myo3a*) and Myosin-9 (*Myh9*) in the zebrafish. The matrix metalloproteinase genes included the Metalloproteinase-14 (*Mmp14b*) gene in the zebrafish. The *Myo1g* gene encodes a protein regulating immune response [206], the *Myo3a* gene encodes an uncharacterized protein, the *Myh9* gene encodes a protein involved in embryonic development [207], and the *Mmp14b* gene encodes an enzyme regulating cell migration during zebrafish gastrulation [208]. The *Myo1g, Myh9* and *Mmp14b* transcripts began to increase right after death and reached abundance maxima at 24 h postmortem, while the *Myo3a* transcript reached an abundance maximum at 12 h postmortem. The significance of these results is twofold: (i) two different technologies (RT-RTqPCR and Gene Meter) have now demonstrated increased transcript abundances and these increases have now been reported in three organisms (human, zebrafish and mouse); and (ii) there might be significant overlap in gene transcripts that increase in abundance in death as we have showed with myosin- and matrix metalloprotease genes, which warrants further studies using other vertebrates. The purpose of such studies would be to understand common mechanisms involved in the shutdown of highly ordered biological systems.

### 4.4. Stability of cell types

Changes of the cell types in the samples with postmortem times could also account for the increases in the transcript abundances with postmortem time because of the differential survival of various cell types. In the case of human blood cells, for example, eosinophils, monocytes, neutrophils and lymphocytes were present just after death, but at 60 h postmortem eosinophils and monocytes were not found, at 66 h postmortem neutrophils were not found and at 86 h postmortem lymphocytes were not found [209]. Skeletal muscle stem cells from mouse, for example, adopt a dormant cell state and retain regenerative capacity for 14–17 days after organismal death [210]. Fibroblast cells from sheep can be cultured for 56 h [211], and fibroblast cells from goats can be cultured for 41–160 days postmortem [212,213]. Similarly, inner ear stem cells from mice can be cultured after 5–10 days postmortem [214]. Taken together, some cells are more resilient than others and are the last ones to die. If stem cells are the last ones to die, then the global transcriptome will become more ‘stem-like’ with postmortem time.

If the cellular composition of the organ/tissues did change between sampling times, then this could contribute to the increases in transcript abundances. Our study analysed whole zebrafish and dissected organs of the mice that contain multiple cell types, which could dilute the transcriptional contribution of any one specific cell type. Current limitations prevent us from using cell-type-specific approaches in systematically analysing the postmortem transcriptome. Furthermore, the number of zebrafish and mouse samples analysed so far is not sufficient to investigate the full magnitude of transcriptional changes that could be occurring.

### 4.5. What do the increases in postmortem transcript abundances mean in the context of life?

Since increases in postmortem transcript abundances occurred in both the zebrafish and the mouse in our study, it is reasonable to suggest that other multicellular eukaryotes will display a similar phenomenon. What does this phenomenon mean in the context of organismal life? We conjecture that the highly ordered structure of an organism—evolved and refined through natural selection and self-organizing processes [215]—undergoes a thermodynamically driven process of spontaneous disintegration through complex pathways, which apparently involve the increased abundance of specific gene transcripts and putative feedback loops. While evolution played a role in pre-patterning of these pathways, it probably does not play any role in its disintegration fate. However, one could argue that some of these pathways have evolved to favour healing or ‘resuscitation’ after severe injury, which would be a possible adaptive advantage. The increased abundance of inflammation response transcripts, for example, putatively indicates that a signal of infection or injury is sensed by the still alive cells after death of the body. Alternatively, these increases could be due to fast decay of some repressors of genes or whole pathways leading to the transcription of genes. Hence, it will be of interest to study this in more detail, since this could, for example, provide insights into how to better preserve organs retrieved for transplantation.

We observed clear qualitative and quantitative differences between two organs (liver and brain) in the mouse in their degradation profiles (figure 2). We also showed an increase in transcript abundances for immunity, inflammation and cancer genes within 1 h of death (figure 11). It would be interesting to explore if these differences are comparable to what occurs in humans, and we wonder how much of the transplant success could be attributed to differences in the synchronicity of postmortem expression profiles rather than immunosuppression agents [216,217]. Our study provides an alternative perspective to the fate of transplant recipients due to the increase of transcripts of regulatory and response genes after the sample has been harvested from the donor.

## 5. Conclusion

This is the first comprehensive study to assess changes in transcriptomic profiles after organismal death and raises interesting questions relative to transplantology, inflammation, cancer, evolution and molecular biology.

## Supplementary Material

Fig S1; Fig S2; Table S1; Table S2; Table S3

## Data accessibility

The datasets supporting this article have been uploaded as part of the electronic supplementary material (see additional files) and are available at the Dryad Digital Repository, http://dx.doi.org/10.5061/dryad.hv223 [218].

## References

[1] Monod J 1961 Genetic regulatory mechanisms in the synthesis of proteins. J. Mol. Biol. 3, 318–356. (doi:10.1016/S0022-2836(61)80072-7) 13718526 10.1016/s0022-2836(61)80072-7

[2] Zong WX, Thompson CB 2006 Necrotic death as a cell fate. Genes Dev. 20, 1–15. (doi:10.1101/gad.1376506) 16391229 10.1101/gad.1376506

[3] Galluzzi L, Bravo-San Pedro JM, Kroemer G 2014 Organelle-specific initiation of cell death. Nat. Cell. Biol. 16, 728–736. (doi:10.1038/ncb3005) 25082195 10.1038/ncb3005

[4] Ferri KF, Kroemer G 2001 Organelle-specific initiation of cell death pathways. Nat. Cell. Biol. 3, E255–E263. (doi:10.1038/ncb1101-e255) 11715037 10.1038/ncb1101-e255

[5] Syntichaki P, Tavernarakis N 2002 Death by necrosis. Uncontrollable catastrophe, or is there order behind the chaos? EMBO Rep. 3, 604–609. (doi:10.1093/embo-reports/kvf138) 12101090 10.1093/embo-reports/kvf138 PMC1084192

[6] Pozhitkov AE, Noble PA, Bryk J, Tautz D 2014 A revised design for microarray experiments to account for experimental noise and uncertainty of probe response. PLoS ONE 9, e91295 (doi:10.1371/journal.pone.0091295) 24618910 10.1371/journal.pone.0091295 PMC3949741

[7] Harrison A et al. 2013 Physico-chemical foundations underpinning microarray and next-generation sequencing experiments. Nucleic Acids Res. 41, 2779–2796. (doi:10.1093/nar/gks1358) 23307556 10.1093/nar/gks1358 PMC3597649

[8] Hunter MC, Pozhitkov AE, Noble PA In press. Microbial signatures of oral dysbiosis, periodontitis and edentulism revealed by Gene Meter methodology. J. Microbiol. Methods 131, 85–101. (doi:10.1101/070367) 10.1016/j.mimet.2016.09.019 27717873

[9] Hunter MC, Pozhitkov AE, Noble PA In press. Accurate predictions of postmortem interval using linear regression analyses of Gene Meter expression data. (doi:10.1101/058370) 10.1016/j.forsciint.2017.02.027 28329724

[10] Domazet-Loso T, Tautz D 2010 Phylostratigraphic tracking of cancer genes suggests a link to the emergence of multicellularity in metazoa. BMC Biol. 8, 66 (doi:10.1186/1741-7007-8-66) 20492640 10.1186/1741-7007-8-66 PMC2880965

[11] Seear PJ, Sweeney GE 2008 Stability of RNA isolated from post-mortem tissues of Atlantic salmon (*Salmo salar* L.). Fish Physiol. Biochem. 34, 19–24. (doi:10.1007/s10695-007-9141-x) 18649019 10.1007/s10695-007-9141-x

[12] Bahar B, Monahan FJ, Moloney AP, Schmidt O, MacHugh DE, Sweeney T 2007 Long-term stability of RNA in post-mortem bovine skeletal muscle, liver and subcutaneous adipose tissues. BMC Mol. Biol. 8, 108 (doi:10.1186/1471-2199-8-108) 18047648 10.1186/1471-2199-8-108 PMC2234424

[13] Inoue H, Kimura A, Tuji T 2002 Degradation profile of mRNA in a dead rat body: basic semi-quantification study. Forensic Sci. Int. 130, 127–132. (doi:10.1016/S0379-0738(02)00352-3) 12477633 10.1016/s0379-0738(02)00352-3

[14] Johnson SA, Morgan DG, Finch CE 1986 Extensive postmortem stability of RNA from rat and human brain. J. Neurosci. Res. 16, 267–280. (doi:10.1002/jnr.490160123) 2427740 10.1002/jnr.490160123

[15] Catts VS, Catts SV, Fernandez HR, Taylor JM, Coulson EJ, Lutze-Mann LH 2005 A microarray study of post-mortem mRNA degradation in mouse brain tissue. Brain Res. Mol. Brain Res. 138, 164–177. (doi:10.1016/j.molbrainres.2005.04.017) 15921819 10.1016/j.molbrainres.2005.04.017

[16] Heinrich M, Matt K, Lutz-Bonengel S, Schmidt U 2007 Successful RNA extraction from various human postmortem tissues. Int. J. Legal Med. 121, 136–142. (doi:10.1007/s00414-006-0131-9) 17115174 10.1007/s00414-006-0131-9

[17] Neymotin B, Athanasiadou R, Gresham D 2014 Determination of *in vivo* RNA kinetics using RATE-seq. RNA 20, 1645–1652. (doi:10.1261/rna.045104.114) 25161313 10.1261/rna.045104.114 PMC4174445

[18] Spilka R et al. 2014 eIF3a is over-expressed in urinary bladder cancer and influences its phenotype independent of translation initiation. Cell. Oncol. (Dordr). 37, 253 (doi:10.1007/s13402-014-0181-9) 25070653 10.1007/s13402-014-0181-9 PMC13004439

[19] Bortolotti GR, Tella JL, Baos R, Marchant TA 2007 Stress response during development predicts fitness in a wild, long lived vertebrate. Proc. Natl Acad. Sci. USA 104, 8880–8884. (doi:10.1073/pnas.0700232104) 17517658 10.1073/pnas.0700232104 PMC1868653

[20] Klose MK, Robertson RM 2004 Stress-induced thermoprotection of neuromuscular transmission. Integr. Comp. Biol. 44, 14–20. (doi:10.1093/icb/44.1.14) 21680481 10.1093/icb/44.1.14

[21] Skaggs HS, Xing H, Wilkerson DC, Murphy LA, Hong Y, Mayhew CN, Sarge KD 2007 HSF1-TPR interaction facilitates export of stress-induced HSP70 mRNA. J. Biol. Chem. 282, 33 902–33 907. (doi:10.1074/jbc.M704054200) 10.1074/jbc.M704054200 PMC2266631 17897941

[22] Snow CJ, Paschal BM 2014 Roles of the nucleoporin Tpr in cancer and aging. Adv. Exp. Med. Biol. 773, 309–322. (doi:10.1007/978-1-4899-8032-8_14) 24563354 10.1007/978-1-4899-8032-8_14

[23] David-Watine B 2011 Silencing nuclear pore protein Tpr elicits a senescent-like phenotype in cancer cells. PLoS ONE 6, e22423 (doi:10.1371/journal.pone.0022423) 21811608 10.1371/journal.pone.0022423 PMC3139644

[24] Kadeba PI, Scammel JG, Cioffi DL 2013 The chaperone heat shock protein 90 (Hsp90) participates in the endothelial store operated calcium entry heterocomplex. FASEB J. 27, 724.

[25] Mayer MP, Bukau B 2005 Hsp70 chaperones: cellular functions and molecular mechanism. Cell. Mol. Life Sci. 62, 670–684. (doi:10.1007/s00018-004-4464-6) 15770419 10.1007/s00018-004-4464-6 PMC2773841

[26] Jakobsson ME, Davydova E, Małecki J, Moen A, Falnes PØ 2015 *Saccharomyces cerevisiae* eukaryotic elongation factor 1A (eEF1A) is methylated at Lys-390 by a METTL21-like methyltransferase. PLoS ONE 10, e0131426 (doi:10.1371/journal.pone.0131426.t002) 26115316 10.1371/journal.pone.0131426 PMC4482628

[27] Höhfeld J, Hartl FU 1994 Requirement of the chaperonin cofactor Hsp10 for protein sorting in yeast mitochondria. J. Cell. Biol. 126, 305–315. (doi:10.1083/jcb.126.2.305) 7913473 10.1083/jcb.126.2.305 PMC2200036

[28] Wu BJ, Morimoto RI 1985 Transcription of the human hsp70 gene is induced by serum stimulation. Proc. Natl Acad. Sci. USA 82, 6070–6074. (doi:10.1073/pnas.82.18.6070) 3862119 10.1073/pnas.82.18.6070 PMC390701

[29] Ngr di A, Domoki F, Dgi R, Borda S, Pkski M, Szab A, Bari F 2003 Up-regulation of cerebral carbonic anhydrase by anoxic stress in piglets. J. Neurochem. 85, 843–850. (doi:10.1046/j.1471-4159.2003.01721.x) 12716416 10.1046/j.1471-4159.2003.01721.x

[30] Lin KH et al. 2015 NFIL3 suppresses hypoxia-induced apoptotic cell death by targeting the insulin-like growth factor 2 receptor. J. Cell. Biochem. 116, 1113–1120. (doi:10.1002/jcb.25067) 25536374 10.1002/jcb.25067

[31] Zhang W, Zhang J, Kornuc M, Kwan K, Frank R, Nimer SD 1995 Molecular cloning and characterization of NF-IL3A, a transcriptional activator of the human interleukin-3 promoter. Mol. Cell Biol. 15, 6055–6063. (doi:10.1128/MCB.15.11.6055) 7565758 10.1128/mcb.15.11.6055 PMC230857

[32] Smith TG, Robbins PA, Ratcliffe PJ 2008 The human side of hypoxia-inducible factor. Br. J. Haematol. 141, 325–334. (doi:10.1111/j.1365-2141.2008.07029.x) 18410568 10.1111/j.1365-2141.2008.07029.x PMC2408651

[33] Ino Y, Yamazaki-Itoh R, Oguro S, Shimada K, Kosuge T, Zavada J, Kanai Y, Hiraoka N 2013 Arginase II expressed in cancer-associated fibroblasts indicates tissue hypoxia and predicts poor outcome in patients with pancreatic cancer. PLoS ONE 8, e55146 (doi:10.1371/journal.pone.0055146) 23424623 10.1371/journal.pone.0055146 PMC3570471

[34] Lafleur VN, Richard S, Richard DE 2014 Transcriptional repression of hypoxia-inducible factor-1 (HIF-1) by the protein arginine methyltransferase PRMT1. Mol. Biol. Cell. 25, 925–935. (doi:10.1091/mbc.E13-07-0423) 24451260 10.1091/mbc.E13-07-0423 PMC3952860

[35] Devlin CM et al. 2011 Dihydroceramide-based response to hypoxia. J. Biol. Chem. 286, 38 069–38 078. (doi:10.1074/jbc.M111.297994) 10.1074/jbc.M111.297994 PMC3207482 21914808

[36] Hannun YA, Obeid LM 2008 Principles of bioactive lipid signalling: lessons from sphingolipids. Nat. Rev. Mol. Cell Biol. 9, 139–150. (doi:10.1038/nrm2329) 18216770 10.1038/nrm2329

[37] Mao C, Obeid LM 2008 Ceramidases: regulators of cellular responses mediated by ceramide, sphingosine, and sphingosine-1-phosphate. Biochim. Biophys. Acta 1781, 424–434. (doi:10.1016/j.bbalip.2008.06.002) 18619555 10.1016/j.bbalip.2008.06.002 PMC2614331

[38] Rhee SG, Chae HZ, Kim K 2005 Peroxiredoxins: a historical overview and speculative preview of novel mechanisms and emerging concepts in cell signaling. Free Radic. Biol. Med. 38, 1543–1552. (doi:10.1016/j.freeradbiomed.2005.02.026) 15917183 10.1016/j.freeradbiomed.2005.02.026

[39] Salzano S et al. 2014 Linkage of inflammation and oxidative stress via release of glutathionylated peroxiredoxin-2, which acts as a danger signal. Proc. Natl Acad. Sci. USA 111, 12 157–12 162. (doi:10.1073/pnas.1401712111) 10.1073/pnas.1401712111 PMC4143057 25097261

[40] Landau G et al. 2012 Expression profiling and biochemical analysis suggest stress response as a potential mechanism inhibiting proliferation of polyamine-depleted cells. J. Biol. Chem. 287, 35 825–35 837. (doi:10.1074/jbc.M112.381335) 22942278 10.1074/jbc.M112.381335 PMC3476252

[41] Fornace AJ Jr, Jackman J, Hollander MC, Hoffman-Liebermann B, Liebermann DA 1992 Genotoxic-stress-response genes and growth-arrest genes: gadd, MyD, and other genes induced by treatments eliciting growth arrest. Ann. NY Acad. Sci. 663, 139–153. (doi:10.1111/j.1749-6632.1992.tb38657.x) 1482047 10.1111/j.1749-6632.1992.tb38657.x

[42] Schmitz I 2013 Gadd45 proteins in immunity. In Gadd45 Stress Sensor Genes, Advances in Experimental Medicine and Biology 793 (eds Liebermann DA, Hoffman B). New York, NY: Springer Science+Business Media. 10.1007/978-1-4614-8289-5_4 24104473

[43] Lisowski P, Wieczorek M, Goscik J, Juszczak GR, Stankiewicz AM, Zwierzchowski L, Swiergiel AH 2013 Effects of chronic stress on prefrontal cortex transcriptome in mice displaying different genetic backgrounds. J. Mol. Neurosci. 50, 33–57. (doi:10.1007/s12031-012-9850-1) 22836882 10.1007/s12031-012-9850-1 PMC3622021

[44] Timpl P et al. 1998 Impaired stress response and reduced anxiety in mice lacking a functional corticotropin-releasing hormone receptor 1. Nat. Genet. 19, 162–166. (doi:10.1038/520) 9620773 10.1038/520

[45] Leikauf GD et al. 2013 Functional genomic assessment of phosgene-induced acute lung injury in mice. Am J. Respir. Cell Mol. Biol. 49, 368–383. (doi:10.1165/rcmb.2012-0337OC) 23590305 10.1165/rcmb.2012-0337OC PMC3824050

[46] Nice TJ, Deng W, Coscoy L, Raulet DH 2010 Stress-regulated targeting of the NKG2D ligand Mult1 by a membrane-associated RING-CH family E3 ligase. J. Immunol. 185, 5369–5376. (doi:10.4049/jimmunol.1000247) 20870941 10.4049/jimmunol.1000247 PMC3001296

[47] Kokame K, Agarwala KL, Kato H, Miyata T 2000 Herp, a new ubiquitin-like membrane protein induced by endoplasmic reticulum stress. J. Biol. Chem. 275, 32 846–32 853. (doi:10.1074/jbc.M002063200) 10.1074/jbc.M002063200 10922362

[48] Coates PJ, Nenutil R, McGregor A, Picksley SM, Crouch DH, Hall PA, Wright EG 2001 Mammalian prohibitin proteins respond to mitochondrial stress and decrease during cellular senescence. Exp. Cell. Res. 265, 262–273. (doi:10.1006/excr.2001.5166) 11302691 10.1006/excr.2001.5166

[49] Wehner KA, Schtz S, Sarnow P 2010 OGFOD1, a novel modulator of eukaryotic translation initiation factor 2alpha phosphorylation and the cellular response to stress. Mol. Cell. Biol. 30, 2006–2016. (doi:10.1128/MCB.01350-09) 20154146 10.1128/MCB.01350-09 PMC2849474

[50] Rock KL, Lai JJ, Kono H 2011 Innate and adaptive immune responses to cell death. Immunol. Rev. 243, 191–205. (doi:10.1111/j.1600-065X.2011.01040.x) 21884177 10.1111/j.1600-065X.2011.01040.x PMC3170128

[51] Crist SA, Elzey BD, Ahmann MT, Ratliff TL 2013 Early growth response-1 (EGR-1) and nuclear factor of activated T cells (NFAT) cooperate to mediate CD40L expression in megakaryocytes and platelets. J. Biol. Chem. 288, 33 985–33 996. (doi:10.1074/jbc.M113.511881) 24106272 10.1074/jbc.M113.511881 PMC3837138

[52] Li S, Miao T, Sebastian M, Bhullar P, Ghaffari E, Liu M, Symonds AL, Wang P 2012 The transcription factors Egr2 and Egr3 are essential for the control of inflammation and antigen-induced proliferation of B and T cells. Immunity 37, 685–696. (doi:10.1016/j.immuni.2012.08.001) 23021953 10.1016/j.immuni.2012.08.001 PMC3477314

[53] Jayaraman P, Sada-Ovalle I, Nishimura T, Anderson AC, Kuchroo VK, Remold HG, Behar SM 2013 IL-1β promotes antimicrobial immunity in macrophages by regulating TNFR signaling and caspase-3 activation. J. Immunol. 190, 4196–4204. (doi:10.4049/jimmunol.1202688) 23487424 10.4049/jimmunol.1202688 PMC3622150

[54] Hughes AL 2010 Origin and diversification of the L-amino oxidase family in innate immune defenses of animals. Immunogenetics 62, 753–759. (doi:10.1007/s00251-010-0482-8) 20878154 10.1007/s00251-010-0482-8 PMC3004525

[55] Ramirez-Carrozzi V et al. 2011 IL-17C regulates the innate immune function of epithelial cells in an autocrine manner. Nat. Immunol. 12, 1159–1166. (doi:10.1038/ni.2156) 21993848 10.1038/ni.2156

[56] Zuccolo J, Bau J, Childs SJ, Goss GG, Sensen CW, Deans JP 2010 Phylogenetic analysis of the MS4A and TMEM176 gene families. PLoS ONE 5, e9369 (doi:10.1371/journal.pone.0009369) 20186339 10.1371/journal.pone.0009369 PMC2826416

[57] Allen A, Hutton DA, Pearson JP 1998 The MUC2 gene product: a human intestinal mucin. Int. J. Biochem. Cell Biol. 30, 797–801. (doi:10.1016/S1357-2725(98)00028-4) 9722984 10.1016/s1357-2725(98)00028-4

[58] Michelucci A et al. 2013 Immune-responsive gene 1 protein links metabolism to immunity by catalyzing itaconic acid production. Proc. Natl Acad. Sci. USA 110, 7820–7825. (doi:10.1073/pnas.1218599110) 23610393 10.1073/pnas.1218599110 PMC3651434

[59] Honke N et al. 2011 Enforced viral replication activates adaptive immunity and is essential for the control of a cytopathic virus. Nat. Immunol. 13, 51–57. (doi:10.1038/ni.2169) 22101728 10.1038/ni.2169

[60] Martínez-López M, Iborra S, Conde-Garrosa R, Sancho D 2015 Batf3-dependent CD103+ dendritic cells are major producers of IL-12 that drive local Th1 immunity against *Leishmania major* infection in mice. Eur. J. Immunol. 45, 119–129. (doi:10.1002/eji.201444651) 25312824 10.1002/eji.201444651 PMC4316187

[61] Imai T, Baba M, Nishimura M, Kakizaki M, Takagi S, Yoshie O 1997 The T cell-directed CC chemokine TARC is a highly specific biological ligand for CC chemokine receptor 4. J. Biol. Chem. 272, 15 036–15 042. (doi:10.1074/jbc.272.23.15036) 10.1074/jbc.272.23.15036 9169480

[62] Vong QP, Leung WH, Houston J, Li Y, Rooney B, Holladay M, Oostendorp RA, Leung W 2014 TOX2 regulates human natural killer cell development by controlling T-BET expression. Blood 124, 3905–3913. (doi:10.1182/blood-2014-06-582965) 25352127 10.1182/blood-2014-06-582965 PMC4282154

[63] Takaori A 2005 Antiviral defense by APOBEC3 family proteins. Uirusu 55, 267–272. (doi:10.2222/jsv.55.267) 16557012 10.2222/jsv.55.267

[64] Arming S, Wipfler D, Mayr J, Merling A, Vilas U, Schauer R, Schwartz-Albiez R, Vlasak R 2011 The human Cas1 protein: a sialic acid-specific O-acetyltransferase? Glycobiology 21, 553–564. (doi:10.1093/glycob/cwq153) 20947662 10.1093/glycob/cwq153 PMC7108626

[65] Crocker PR, Paulson JC, Varki A 2007 Siglecs and their roles in the immune system. Nat. Rev. Immunol. 7, 255–266. (doi:10.1038/nri2056) 17380156 10.1038/nri2056

[66] Crocker PR, Varki A 2001 Siglecs in the immune system. Immunology 103, 137–145. (doi:10.1046/j.0019-2805.2001.01241.x) 11412300 10.1046/j.0019-2805.2001.01241.x PMC1783234

[67] Shi WX, Chammas R, Varki NM, Powell L, Varki A 1996 Sialic acid 9-O-acetylation on murine erythroleukemia cells affects complement activation, binding to I-type lectins, and tissue homing. J. Biol. Chem. 271, 31 526–31 532. (doi:10.1074/jbc.271.49.31526) 10.1074/jbc.271.49.31526 8940168

[68] Praper T, Sonnen A, Viero G, Kladnik A, Froelich CJ, Anderluh G, Dalla Serra M, Gilbert RJ 2011 Human perforin employs different avenues to damage membranes. J. Biol. Chem. 286, 2946–2955. (doi:10.1074/jbc.M110.169417) 20889983 10.1074/jbc.M110.169417 PMC3024789

[69] Tschopp J, Masson D, Stanley KK 1986 Structural/functional similarity between proteins involved in complement- and cytotoxic T-lymphocyte-mediated cytolysis. Nature 322, 831–834. (doi:10.1038/322831a0) 2427956 10.1038/322831a0

[70] White SH, Wimley WC, Selsted ME 1995 Structure, function, and membrane integration of defensins. Curr. Opin. Struct. Biol. 5, 521–527. (doi:10.1016/0959-440X(95)80038-7) 8528769 10.1016/0959-440x(95)80038-7

[71] Blanchong CA, Chung EK, Rupert KL, Yang Y, Yang Z, Zhou B, Moulds JM, Yu CY 2001 Genetic, structural and functional diversities of human complement components C4A and C4B and their mouse homologues, Slp and C4. Int. Immunopharmacol. 1, 365–392. (doi:10.1016/S1567-5769(01)00019-4) 11367523 10.1016/s1567-5769(01)00019-4

[72] Chen L, Carico Z, Shih HY, Krangel MS 2015 A discrete chromatin loop in the mouse Tcra-Tcrd locus shapes the TCRδ and TCRα repertoires. Nat. Immunol. 16, 1085–1093. (doi:10.1038/ni.3232) 26258942 10.1038/ni.3232 PMC4575630

[73] Grage-Griebenow E, Flad HD, Ernst M, Bzowska M, Skrzeczyska J, Pryjma J 2000 Human MO subsets as defined by expression of CD64 and CD16 differ in phagocytic activity and generation of oxygen intermediates. Immunobiology 202, 42–50. (doi:10.1016/S0171-2985(00)80051-0) 10879688 10.1016/S0171-2985(00)80051-0

[74] Al-Banna NA, Vaci M, Slauenwhite D, Johnston B, Issekutz TB 2014 CCR4 and CXCR3 play different roles in the migration of T cells to inflammation in skin, arthritic joints, and lymph nodes. Eur. J. Immunol. 44, 1633–1643. (doi:10.1002/eji.201343995) 24700244 10.1002/eji.201343995

[75] Ikutani M et al. 2012 Identification of innate IL-5-producing cells and their role in lung eosinophil regulation and antitumor immunity. J. Immunol. 188, 703–713. (doi:10.4049/jimmunol.1101270) 22174445 10.4049/jimmunol.1101270

[76] Lillard JW Jr, Boyaka PN, Taub DD, McGhee JR 2001 RANTES potentiates antigen-specific mucosal immune responses. J. Immunol. 166, 162–169. (doi:10.4049/jimmunol.166.1.162) 11123289 10.4049/jimmunol.166.1.162

[77] Kawasaki N, Rademacher C, Paulson JC 2011 CD22 regulates adaptive and innate immune responses of B cells. J. Innate Immun. 3, 411–419. (doi:10.1159/000322375) 21178327 10.1159/000322375 PMC3130896

[78] Liu H, Thaker YR, Stagg L, Schneider H, Ladbury JE, Rudd CE 2013 SLP-76 sterile α motif (SAM) and individual H5 α helix mediate oligomer formation for microclusters and T-cell activation. J. Biol. Chem. 288, 29 539–29 549. (doi:10.1074/jbc.M112.4248460) 10.1074/jbc.M112.424846 PMC3795252 23935094

[79] Feeley EM et al. 2011 IFITM3 inhibits influenza A virus infection by preventing cytosolic entry. PLoS Pathog. 7, e1002337 (doi:10.1371/journal.ppat.1002337) 22046135 10.1371/journal.ppat.1002337 PMC3203188

[80] Saben J, Zhong Y, Gomez-Acevedo H, Thakali KM, Borengasser SJ, Andres A, Shankar K 2013 Early growth response protein-1 mediates lipotoxicity-associated placental inflammation: role in maternal obesity. Am. J. Physiol. Endocrinol. Metab. 305, e1–14. (doi:10.1152/ajpendo.00076.2013) 23632636 10.1152/ajpendo.00076.2013 PMC4116409

[81] Ren K, Torres R 2009 Role of interleukin-1beta during pain and inflammation. Brain Res. Rev. 60, 57–64. (doi:10.1016/j.brainresrev.2008.12.020) 19166877 10.1016/j.brainresrev.2008.12.020 PMC3076185

[82] Chen GY, Nuñez G 2010 Sterile inflammation: sensing and reacting to damage. Nat. Rev. Immunol. 10, 826–837. (doi:10.1038/nri2873) 21088683 10.1038/nri2873 PMC3114424

[83] Sedger LM, McDermott MF 2014 TNF and TNF-receptors: from mediators of cell death and inflammation to therapeutic giants – past, present and future. Cytokine Growth Factor Rev. 25, 453–472. (doi:10.1016/j.cytogfr.2014.07.016) 25169849 10.1016/j.cytogfr.2014.07.016

[84] Lee TS, Chau LY 2002 Heme oxygenase-1 mediates the anti-inflammatory effect of interleukin-10 in mice. Nat. Med. 8, 240–246. (doi:10.1038/nm0302-240) 11875494 10.1038/nm0302-240

[85] Piantadosi CA, Withers CM, Bartz RR, MacGarvey NC, Fu P, Sweeney TE, Welty-Wolf KE, Suliman HB 2011 Heme oxygenase-1 couples activation of mitochondrial biogenesis to anti-inflammatory cytokine expression. J. Biol. Chem. 286, 16 374–16 385. (doi:10.1074/jbc.M110.207738) 10.1074/jbc.M110.207738 PMC3091243 21454555

[86] Guo Y et al. 2011 Identification of the orphan G protein-coupled receptor GPR31 as a receptor for 12-(S)-hydroxyeicosatetraenoic acid. J. Biol. Chem. 286, 33 832–33 840. (doi:10.1074/jbc.M110.216564) 10.1074/jbc.M110.216564 PMC3190773 21712392

[87] Bickel M 1993 The role of interleukin-8 in inflammation and mechanisms of regulation. J. Periodontol. 64, 456–460. 8315568

[88] Alcock J, Franklin ML, Kuzawa CW 2012 Nutrient signaling: evolutionary origins of the immune-modulating effects of dietary fat. Q. Rev. Biol. 87, 187–223. (doi:10.1086/666828) 22970557 10.1086/666828

[89] Pearson G, Robinson F, Beers Gibson T, Xu BE, Karandikar M, Berman K, Cobb MH 2001 Mitogen-activated protein (MAP) kinase pathways: regulation and physiological functions. Endocr. Rev. 22, 153–183. (doi:10.1210/edrv.22.2.0428) 11294822 10.1210/edrv.22.2.0428

[90] Tsatsanis C, Androulidaki A, Dermitzaki E, Gravanis A, Margioris AN 2007 Corticotropin releasing factor receptor 1 (CRF_1_) and CRF_2_ agonists exert an anti-inflammatory effect during the early phase of inflammation suppressing LPS-induced TNF-α release from macrophages via induction of COX-2 and PGE_2_. J. Cell. Physiol. 210, 774–783. (doi:10.1002/jcp.20900) 17117478 10.1002/jcp.20900

[91] Frugier T, Morganti-Kossmann MC, O'Reilly D, McLean CA 2010 *In situ* detection of inflammatory mediators in post mortem human brain tissue after traumatic injury. J. Neurotrauma 27, 497–507. (doi:10.1089/neu.2009.1120) 20030565 10.1089/neu.2009.1120

[92] Sawant DV et al. 2012 Bcl6 controls the Th2 inflammatory activity of regulatory T cells by repressing Gata3 function. J. Immunol. 189, 4759–4769. (doi:10.4049/jimmunol.1201794) 23053511 10.4049/jimmunol.1201794 PMC3490013

[93] Correa-Costa M et al. 2014 Activation of platelet-activating factor receptor exacerbates renal inflammation and promotes fibrosis. Lab Invest. 94, 455–466. (doi:10.1038/labinvest.2013.155) 24492283 10.1038/labinvest.2013.155

[94] Watson RP et al. 2012 Increased prokineticin 2 expression in gut inflammation: role in visceral pain and intestinal ion transport. Neurogastroenterol. Motil. 24, 65–75. (doi:10.1111/j.1365-2982.2011.01804.x) 22050240 10.1111/j.1365-2982.2011.01804.x

[95] Pavlov VA, Wang H, Czura CJ, Friedman SG, Tracey KJ 2003 The cholinergic anti-inflammatory pathway: a missing link in neuroimmunomodulation. Mol. Med. 9, 125–134. 14571320 PMC1430829

[96] Lerdrup M, Holmberg C, Dietrich N, Shaulian E, Herdegen T, Jttel M, Kallunki T 2005 Depletion of the AP-1 repressor JDP2 induces cell death similar to apoptosis. Biochim. Biophys. Acta 1745, 29–37. (doi:10.1016/j.bbamcr.2005.06.008) 16026868 10.1016/j.bbamcr.2005.06.008

[97] Hu W, Xu R, Sun W, Szulc ZM, Bielawski J, Obeid LM, Mao C 2010 Alkaline ceramidase 3 (ACER3) hydrolyzes unsaturated long-chain ceramides, and its down-regulation inhibits both cell proliferation and apoptosis. J. Biol. Chem. 285, 7964–7976. (doi:10.1074/jbc.M109.063586) 20068046 10.1074/jbc.M109.063586 PMC2832947

[98] Preston GA, Lyon TT, Yin Y, Lang JE, Solomon G, Annab L, Srinivasan DG, Alcorta DA, Barrett JC 1996 Induction of apoptosis by c-Fos protein. Mol. Cell Biol. 16, 211–218. (doi:10.1128/MCB.16.1.211) 8524298 10.1128/mcb.16.1.211 PMC230994

[99] Adrain C, Creagh EM, Martin SJ 2001 Apoptosis-associated release of Smac/DIABLO from mitochondria requires active caspases and is blocked by Bcl-2. EMBO J. 20, 6627–6636. (doi:10.1093/emboj/20.23.6627) 11726499 10.1093/emboj/20.23.6627 PMC125329

[100] McNeish IA, Lopes R, Bell SJ, McKay TR, Fernandez M, Lockley M, Wheatley SP, Lemoine NR 2005 Survivin interacts with Smac/DIABLO in ovarian carcinoma cells but is redundant in Smac-mediated apoptosis. Exp. Cell. Res. 302, 69–82. (doi:10.1016/j.yexcr.2004.08.029) 15541727 10.1016/j.yexcr.2004.08.029

[101] Zhao F, Wang Q 2012 The protective effect of peroxiredoxin II on oxidative stress induced apoptosis in pancreatic β-cells. Cell. Biosci. 2, 22 (doi:10.1186/2045-3701-2-22) 22709359 10.1186/2045-3701-2-22 PMC3461449

[102] Wu X, Hernandez-Enriquez B, Banas M, Xu R, Sesti F 2013 Molecular mechanisms underlying the apoptotic effect of KCNB1 K^+^ channel oxidation. J. Biol. Chem. 288, 4128–4134. (doi:10.1074/jbc.M112.440933) 23275378 10.1074/jbc.M112.440933 PMC3567663

[103] Tang D, Lahti JM, Kidd VJ 2000 Caspase-8 activation and Bid cleavage contribute to MCF7 cellular execution in a caspase-3-dependent manner during staurosporine-mediated apoptosis. J. Biol. Chem. 275, 9303–9307. (doi:10.1074/jbc.275.13.9303) 10734071 10.1074/jbc.275.13.9303

[104] Chinnadurai G, Vijayalingam S, Rashmi R 2008 BIK, the founding member of the BH3-only family proteins: mechanisms of cell death and role in cancer and pathogenic processes. Oncogene 27, S20–S29. (doi:10.1038/onc.2009.40) 19641504 10.1038/onc.2009.40 PMC2928562

[105] Iwasa H, Kudo T, Maimaiti S, Ikeda M, Maruyama J, Nakagawa K, Hata Y 2013 The RASSF6 tumor suppressor protein regulates apoptosis and the cell cycle via MDM2 protein and p53 protein. J. Biol. Chem. 288, 30 320–30 329. (doi:10.1074/jbc.M113.5073840) 10.1074/jbc.M113.507384 PMC3798497 24003224

[106] Liu X, Dong C, Jiang Z, Wu WK, Chan MT, Zhang J, Li H, Qin K, Sun X 2015 MicroRNA-10b downregulation mediates acute rejection of renal allografts by derepressing BCL2L11. Exp. Cell. Res. 333, 155–163. (doi:10.1016/j.yexcr.2015.01.018) 25659925 10.1016/j.yexcr.2015.01.018

[107] Sayed M, Pelech S, Wong C, Marotta A, Salh B 2001 Protein kinase CK2 is involved in G2 arrest and apoptosis following spindle damage in epithelial cells. Oncogene 20, 6994–7005. (doi:10.1038/sj.onc.1204894) 11704824 10.1038/sj.onc.1204894

[108] Maddigan A et al. 2011 EphB receptors trigger Akt activation and suppress Fas receptor-induced apoptosis in malignant T lymphocytes. J. Immunol. 187, 5983–5994. (doi:10.4049/jimmunol.1003482) 22039307 10.4049/jimmunol.1003482

[109] Okamoto S, Krainc D, Sherman K, Lipton SA 2000 Antiapoptotic role of the p38 mitogen-activated protein kinase–myocyte enhancer factor 2 transcription factor pathway during neuronal differentiation. Proc. Natl Acad. Sci. USA 97, 7561–7566. (doi:10.1073/pnas.130502697) 10852968 10.1073/pnas.130502697 PMC16585

[110] Chiorazzi M et al. 2013 Related F-box proteins control cell death in *Caenorhabditis elegans* and human lymphoma. Proc. Natl Acad. Sci. USA 110, 3943–3948. (doi:10.1073/pnas.1217271110) 23431138 10.1073/pnas.1217271110 PMC3593917

[111] Bloch DB, Nakajima A, Gulick T, Chiche JD, Orth D, de La Monte SM, Bloch KD 2000 Sp110 localizes to the PML-Sp100 nuclear body and may function as a nuclear hormone receptor transcriptional coactivator. Mol. Cell Biol. 20, 6138–6146. (doi:10.1128/MCB.20.16.6138-6146.2000) 10913195 10.1128/mcb.20.16.6138-6146.2000 PMC86089

[112] Bengoechea-Alonso MT, Ericsson J 2010 Tumor suppressor Fbxw7 regulates TGFβ signaling by targeting TGIF1 for degradation. Oncogene 29, 5322–5328. (doi:10.1038/onc.2010.278) 20622901 10.1038/onc.2010.278

[113] Ma Y, Wang B, Li W, Ying G, Fu L, Niu R, Gu F 2010 Reduction of intersectin1-s induced apoptosis of human glioblastoma cells. Brain Res. 1351, 222–228. (doi:10.1016/j.brainres.2010.05.028) 20493827 10.1016/j.brainres.2010.05.028

[114] Gnesutta N, Qu J, Minden A 2001 The serine/threonine kinase PAK4 prevents caspase activation and protects cells from apoptosis. J. Biol. Chem. 276, 14 414–14 419. (doi:10.1074/jbc.M103454200) 10.1074/jbc.M011046200 11278822

[115] Markovich D 2001 Physiological roles and regulation of mammalian sulfate transporters. Physiol. Rev. 81, 1499–1533. 11581495 10.1152/physrev.2001.81.4.1499

[116] Guasti L et al. 2008 Identification of a posttranslational mechanism for the regulation of hERG1 K^+^ channel expression and hERG1 current density in tumor cells. Mol. Cell. Biol. 28, 5043–5060. (doi:10.1128/MCB.00304-08) 18559421 10.1128/MCB.00304-08 PMC2519704

[117] Bremser M et al. 1999 Coupling of coat assembly and vesicle budding to packaging of putative cargo receptors. Cell 96, 495–506. (doi:10.1016/S0092-8674(00)80654-6) 10052452 10.1016/s0092-8674(00)80654-6

[118] Zhen Y, Srensen V, Skjerpen CS, Haugsten EM, Jin Y, Wlchli S, Olsnes S, Wiedlocha A 2012 Nuclear import of exogenous FGF1 requires the ER-protein LRRC59 and the importins Kpnα1 and Kpnβ1. Traffic 13, 650–664. (doi:10.1111/j.1600-0854.2012.01341.x) 22321063 10.1111/j.1600-0854.2012.01341.x

[119] Bangs P, Burke B, Powers C, Craig R, Purohit A, Doxsey S 1998 Functional analysis of Tpr: identification of nuclear pore complex association and nuclear localization domains and a role in mRNA export. J. Cell. Biol. 143, 1801–1812. (doi:10.1083/jcb.143.7.1801) 9864356 10.1083/jcb.143.7.1801 PMC2175216

[120] Nakielny S, Siomi MC, Siomi H, Michael WM, Pollard V, Dreyfuss G 1996 Transportin: nuclear transport receptor of a novel nuclear protein import pathway. Exp. Cell. Res. 229, 261–266. (doi:10.1006/excr.1996.0369) 8986607 10.1006/excr.1996.0369

[121] Chi NC, Adam EJ, Adam SA 1995 Sequence and characterization of cytoplasmic nuclear protein import factor p97. J. Cell. Biol. 130, 265–274. (doi:10.1083/jcb.130.2.265) 7615630 10.1083/jcb.130.2.265 PMC2199936

[122] Tang BL, Low DY, Tan AE, Hong W 1998 Syntaxin 10: a member of the syntaxin family localized to the trans-Golgi network. Biochem. Biophys. Res. Commun. 242, 345–350. (doi:10.1006/bbrc.1997.7966) 9446797 10.1006/bbrc.1997.7966

[123] Chen G 2014 Biochemical properties of urea transporters. Subcell. Biochem. 73, 109–126. (doi:10.1007/978-94-017-9343-8_7) 25298341 10.1007/978-94-017-9343-8_7

[124] Palmieri L et al. 2001 Citrin and aralar1 are Ca^2+^-stimulated aspartate/glutamate transporters in mitochondria. EMBO J. 20, 5060–5069. (doi:10.1093/emboj/20.18.5060) 11566871 10.1093/emboj/20.18.5060 PMC125626

[125] Hatanaka T, Huang W, Ling R, Prasad PD, Sugawara M, Leibach FH, Ganapathy V 2001 Evidence for the transport of neutral as well as cationic amino acids by ATA3, a novel and liver-specific subtype of amino acid transport system A. Biochim. Biophys. Acta 1510, 10–17. (doi:10.1016/S0005-2736(00)00390-4) 11342143 10.1016/s0005-2736(00)00390-4

[126] Conchon S, Cao X, Barlowe C, Pelham HR 1999 Got1p and Sft2p: membrane proteins involved in traffic to the Golgi complex. EMBO J. 18, 3934–3946. (doi:10.1093/emboj/18.14.3934) 10406798 10.1093/emboj/18.14.3934 PMC1171469

[127] Hautbergue GM, Hung ML, Walsh MJ, Snijders AP, Chang CT, Jones R, Ponting CP, Dickman MJ, Wilson SA 2009 UIF, a New mRNA export adaptor that works together with REF/ALY, requires FACT for recruitment to mRNA. Curr. Biol. 19, 1918–1924. (doi:10.1016/j.cub.2009.09.041) 19836239 10.1016/j.cub.2009.09.041 PMC2828547

[128] Grempler R, Augustin R, Froehner S, Hildebrandt T, Simon E, Mark M, Eickelmann P 2012 Functional characterisation of human SGLT-5 as a novel kidney-specific sodium-dependent sugar transporter. FEBS Lett. 586, 248–253. (doi:10.1016/j.febslet.2011.12.027) 22212718 10.1016/j.febslet.2011.12.027

[129] Dietmeier K, Hönlinger A, Bömer U, Dekker PJ, Eckerskorn C, Lottspeich F, Kübrich M, Pfanner N 1997 Tom5 functionally links mitochondrial preprotein receptors to the general import pore. Nature 388, 195–200. (doi:10.1038/40663) 9217162 10.1038/40663

[130] Annilo T et al. 2002 Identification and characterization of a novel ABCA subfamily member, ABCA12, located in the lamellar ichthyosis region on 2q34. Cytogenet. Genome Res. 98, 169–176. (doi:10.1159/000069811) 12697999 10.1159/000069811

[131] Moraes LA, Piqueras L, Bishop-Bailey D 2006 Peroxisome proliferator-activated receptors and inflammation. Pharmacol. Ther. 110, 371–385. (doi:10.1016/j.pharmthera.2005.08.007) 16168490 10.1016/j.pharmthera.2005.08.007

[132] Allikmets R, Gerrard B, Hutchinson A, Dean M 1996 Characterization of the human ABC superfamily: isolation and mapping of 21 new genes using the expressed sequence tags database. Hum. Mol. Genet. 5, 1649–1655. (doi:10.1093/hmg/5.10.1649) 8894702 10.1093/hmg/5.10.1649

[133] Chew CS, Parente JA Jr, Chen X, Chaponnier C, Cameron RS 2000 The LIM and SH3 domain-containing protein, lasp-1, may link the cAMP signaling pathway with dynamic membrane restructuring activities in ion transporting epithelia. J. Cell. Sci. 113, 2035–2045. 10806114 10.1242/jcs.113.11.2035

[134] Phillips-Krawczak CA et al. 2015 COMMD1 is linked to the WASH complex and regulates endosomal trafficking of the copper transporter ATP7A. Mol. Biol. Cell. 26, 91–103. (doi:10.1091/mbc.E14-06-1073) 25355947 10.1091/mbc.E14-06-1073 PMC4279232

[135] Tyson JR, Stirling CJ 2000 LHS1 and SIL1 provide a lumenal function that is essential for protein translocation into the endoplasmic reticulum. EMBO J. 19, 6440–6452. (doi:10.1093/emboj/19.23.6440) 11101517 10.1093/emboj/19.23.6440 PMC305876

[136] Sirrenberg C, Bauer MF, Guiard B, Neupert W, Brunner M 1996 Import of carrier proteins into the mitochondrial inner membrane mediated by Tim22. Nature 384, 582–585. (doi:10.1038/384582a0) 8955274 10.1038/384582a0

[137] Rubino M, Miaczynska M, Lipp R, Zerial M 2000 Selective membrane recruitment of EEA1 suggests a role in directional transport of clathrin-coated vesicles to early endosomes. J. Biol. Chem. 275, 3745–3748. (doi:10.1074/jbc.275.6.3745) 10660521 10.1074/jbc.275.6.3745

[138] Zhu XR, Netzer R, Bhlke K, Liu Q, Pongs O 1999 Structural and functional characterization of Kv6.2 a new gamma-subunit of voltage-gated potassium channel. Receptors Channels 6, 337–350. 10551266

[139] Toyama R, Kobayashi M, Tomita T, Dawid IB 1998 Expression of LIM-domain binding protein (ldb) genes during zebrafish embryogenesis. Mech. Dev. 71, 197–200. (doi:10.1016/S0925-4773(97)00202-5) 9507128 10.1016/s0925-4773(97)00202-5

[140] Kida YS, Sato T, Miyasaka KY, Suto A, Ogura T 2007 Daam1 regulates the endocytosis of EphB during the convergent extension of the zebrafish notochord. Proc. Natl Acad. Sci. USA 104, 6708–6713. (doi:10.1073/pnas.0608946104) 17412835 10.1073/pnas.0608946104 PMC1871850

[141] Gilpin BJ, Loechel F, Mattei MG, Engvall E, Albrechtsen R, Wewer UM 1998 A novel, secreted form of human ADAM 12 (meltrin alpha) provokes myogenesis in vivo. J. Biol. Chem. 273, 157–166. (doi:10.1074/jbc.273.1.157) 9417060 10.1074/jbc.273.1.157

[142] Sano K, Inohaya K, Kawaguchi M, Yoshizaki N, Iuchi I, Yasumasu S 2008 Purification and characterization of zebrafish hatching enzyme – an evolutionary aspect of the mechanism of egg envelope digestion. FEBS J. 275, 5934–5946. (doi:10.1111/j.1742-4658.2008.06722.x) 19021768 10.1111/j.1742-4658.2008.06722.x

[143] Hofmeister-Brix A, Kollmann K, Langer S, Schultz J, Lenzen S, Baltrusch S 2013 Identification of the ubiquitin-like domain of midnolin as a new glucokinase interaction partner. J. Biol. Chem. 288, 35 824–35 839. (doi:10.1074/jbc.M113.526632) 10.1074/jbc.M113.526632 PMC3861633 24187134

[144] Tsukahara M, Suemori H, Noguchi S, Ji ZS, Tsunoo H 2000 Novel nucleolar protein, midnolin, is expressed in the mesencephalon during mouse development. Gene 254, 45–55. (doi:10.1016/S0378-1119(00)00259-6) 10974535 10.1016/s0378-1119(00)00259-6

[145] Hong SK, Dawid IB 2009 FGF-dependent left–right asymmetry patterning in zebrafish is mediated by Ier2 and Fibp1. Proc. Natl Acad. Sci. USA 106, 2230–2235. (doi:10.1073/pnas.0812880106) 19164561 10.1073/pnas.0812880106 PMC2650137

[146] Kwong RW, Perry SF 2013 The tight junction protein claudin-b regulates epithelial permeability and sodium handling in larval zebrafish, *Danio rerio*. Am. J. Physiol. Regul. Integr. Comp. Physiol. 304, R504–R513. (doi:10.1152/ajpregu.00385.2012) 23364531 10.1152/ajpregu.00385.2012 PMC3627946

[147] Ing T, Aoki Y 2002 Expression of RGS2, RGS4 and RGS7 in the developing postnatal brain. Eur. J. Neurosci. 15, 929–936. (doi:10.1046/j.1460-9568.2002.01925.x) 11906535 10.1046/j.1460-9568.2002.01925.x

[148] Wu T, Patel H, Mukai S, Melino C, Garg R, Ni X, Chang J, Peng C 2000 Activin, inhibin, and follistatin in zebrafish ovary: expression and role in oocyte maturation. Biol. Reprod. 62, 1585–1592. (doi:10.1095/biolreprod62.6.1585) 10819759 10.1095/biolreprod62.6.1585

[149] Malinauskas T, Aricescu AR, Lu W, Siebold C, Jones EY 2011 Modular mechanism of Wnt signaling inhibition by Wnt inhibitory factor 1. Nat. Struct. Mol. Biol. 18, 886–893. (doi:10.1038/nsmb.2081) 21743455 10.1038/nsmb.2081 PMC3430870

[150] Zagon IS, Wu Y, McLaughlin PJ 1999 Opioid growth factor and organ development in rat and human embryos. Brain Res. 839, 313–322. (doi:10.1016/S0006-8993(99)01753-9) 10519055 10.1016/s0006-8993(99)01753-9

[151] Takano A, Zochi R, Hibi M, Terashima T, Katsuyama Y 2010 Expression of strawberry notch family genes during zebrafish embryogenesis. Dev. Dyn. 239, 1789–1796. (doi:10.1002/dvdy.22287) 20503374 10.1002/dvdy.22287

[152] Pierani A, Moran-Rivard L, Sunshine MJ, Littman DR, Goulding M, Jessell TM 2001 Control of interneuron fate in the developing spinal cord by the progenitor homeodomain protein Dbx1. Neuron 29, 367–384. (doi:10.1016/S0896-6273(01)00212-4) 11239429 10.1016/s0896-6273(01)00212-4

[153] Buonamici S, Chakraborty S, Senyuk V, Nucifora G 2003 The role of EVI1 in normal and leukemic cells. Blood Cells Mol. Dis. 31, 206–212. (doi:10.1016/S1079-9796(03)00159-1) 12972028 10.1016/s1079-9796(03)00159-1

[154] Litwack ED, Babey R, Buser R, Gesemann M, O'Leary DD 2004 Identification and characterization of two novel brain-derived immunoglobulin superfamily members with a unique structural organization. Mol. Cell. Neurosci. 25, 263–274. (doi:10.1016/j.mcn.2003.10.016) 15019943 10.1016/j.mcn.2003.10.016

[155] Gazit R, Mandal PK, Ebina W, Ben-Zvi A, Nombela-Arrieta C, Silberstein LE, Rossi DJ 2014 Fgd5 identifies hematopoietic stem cells in the murine bone marrow. J. Exp. Med. 211, 1315–1331. (doi:10.1084/jem.20130428) 24958848 10.1084/jem.20130428 PMC4076584

[156] Zhang J, Tomasini AJ, Mayer AN 2008 RBM19 is essential for preimplantation development in the mouse. BMC Dev. Biol. 8, 115 (doi:10.1186/1471-213X-8-115) 19087264 10.1186/1471-213X-8-115 PMC2627835

[157] Tang K, Xie X, Park JI, Jamrich M, Tsai S, Tsai MJ 2010 COUP-TFs regulate eye development by controlling factors essential for optic vesicle morphogenesis. Development 137, 725–734. (doi:10.1242/dev.040568) 20147377 10.1242/dev.040568 PMC2827684

[158] Zhang P, Bennoun M, Gogard C, Bossard P, Leclerc I, Kahn A, Vasseur-Cognet M 2002 Expression of COUP-TFII in metabolic tissues during development. Mech. Dev. 119, 109–114. (doi:10.1016/S0925-4773(02)00286-1) 12385758 10.1016/s0925-4773(02)00286-1

[159] Coumailleau P, Duprez D 2009 Sim1 and Sim2 expression during chick and mouse limb development. Int. J. Dev. Biol. 53, 149–157. (doi:10.1387/ijdb.082659pc) 19123137 10.1387/ijdb.082659pc

[160] Kondou H et al. 2013 Sodium-coupled neutral amino acid transporter 4 functions as a regulator of protein synthesis during liver development. Hepatol. Res. 43, 1211–1223. (doi:10.1111/hepr.12069) 23607685 10.1111/hepr.12069

[161] Li Z, Lai G, Deng L, Han Y, Zheng D, Song W 2012 Association of SLC38A4 and system A with abnormal fetal birth weight. Exp. Ther. Med. 3, 309–313. 22969887 10.3892/etm.2011.392 PMC3438638

[162] Tiberi L et al. 2012 BCL6 controls neurogenesis through Sirt1-dependent epigenetic repression of selective Notch targets. Nat. Neurosci. 15, 1627–1635. (doi:10.1038/nn.3264) 23160044 10.1038/nn.3264

[163] Matsuoka RL, Sun LO, Katayama K, Yoshida Y, Kolodkin AL 2013 Sema6B, Sema6C, and Sema6D expression and function during mammalian retinal development. PLoS ONE 8, e63207 (doi:10.1371/journal.pone.0063207) 23646199 10.1371/journal.pone.0063207 PMC3640007

[164] Tamada H, Sakashita E, Shimazaki K, Ueno E, Hamamoto T, Kagawa Y, Endo H 2002 cDNA cloning and characterization of Drb1, a new member of RRM-type neural RNA-binding protein. Biochem. Biophys. Res. Commun. 297, 96–104. (doi:10.1016/S0006-291X(02)02132-0) 12220514 10.1016/s0006-291x(02)02132-0

[165] Rempel RE et al. 2000 Loss of E2F4 activity leads to abnormal development of multiple cellular lineages. Mol. Cell. 6, 293–306. (doi:10.1016/S1097-2765(00)00030-7) 10983977 10.1016/s1097-2765(00)00030-7

[166] Miyares RL, Stein C, Renisch B, Anderson JL, Hammerschmidt M, Farber SA 2013 Long-chain acyl-CoA synthetase 4A regulates Smad activity and dorsoventral patterning in the zebrafish embryo. Dev. Cell. 27, 635–647. (doi:10.1016/j.devcel.2013.11.011) 24332754 10.1016/j.devcel.2013.11.011 PMC3895552

[167] Louryan S, Biermans J, Flemal F 1995 Nerve growth factor in the developing craniofacial region of the mouse embryo. Eur. J. Morphol. 33, 415–419. 8907554

[168] Ko JA, Gondo T, Inagaki S, Inui M 2005 Requirement of the transmembrane semaphorin Sema4C for myogenic differentiation. FEBS Lett. 579, 2236–2242. (doi:10.1016/j.febslet.2005.03.022) 15811348 10.1016/j.febslet.2005.03.022

[169] Wu H et al. 2009 Sema4C expression in neural stem/progenitor cells and in adult neurogenesis induced by cerebral ischemia. J. Mol. Neurosci. 39, 27–39. (doi:10.1007/s12031-009-9177-8) 19189244 10.1007/s12031-009-9177-8

[170] Pathirage NA et al. 2013 Homeobox gene transforming growth factor β-induced factor-1 (TGIF-1) is a regulator of villous trophoblast differentiation and its expression is increased in human idiopathic fetal growth restriction. Mol. Hum. Reprod. 19, 665–675. (doi:10.1093/molehr/gat042) 23761267 10.1093/molehr/gat042

[171] Heger S et al. 2007 Enhanced at puberty 1 (EAP1) is a new transcriptional regulator of the female neuroendocrine reproductive axis. J. Clin Invest. 117, 2145–2154. (doi:10.1172/JCI31752) 17627301 10.1172/JCI31752 PMC1906733

[172] Miao H, Strebhardt K, Pasquale EB, Shen TL, Guan JL, Wang B 2005 Inhibition of integrin-mediated cell adhesion but not directional cell migration requires catalytic activity of EphB3 receptor tyrosine kinase. Role of Rho family small GTPases. J. Biol. Chem. 280, 923–932. (doi:10.1074/jbc.M411383200) 15536074 10.1074/jbc.M411383200

[173] Vogel T, Boettger-Tong H, Nanda I, Dechend F, Agulnik AI, Bishop CE, Schmid M, Schmidtke J 1998 A murine TSPY. Chromosome Res. 6, 35–40. (doi:10.1023/A:1009214307764) 9510508 10.1023/a:1009214307764

[174] Janesick A, Shiotsugu J, Taketani M, Blumberg B 2012 RIPPLY3 is a retinoic acid-inducible repressor required for setting the borders of the pre-placodal ectoderm. Development 139, 1213–1224. (doi:10.1242/dev.071456) 22354841 10.1242/dev.071456 PMC3283127

[175] Zhou L, Zhang Z, Zheng Y, Zhu Y, Wei Z, Xu H, Tang Q, Kong X, Hu L 2011 SKAP2, a novel target of HSF4b, associates with NCK2/F-actin at membrane ruffles and regulates actin reorganization in lens cell. J. Cell. Mol. Med. 15, 783–795. (doi:10.1111/j.1582-4934.2010.01048.x) 20219016 10.1111/j.1582-4934.2010.01048.x PMC3922667

[176] Wittschieben J, Shivji MK, Lalani E, Jacobs MA, Marini F, Gearhart PJ, Rosewell I, Stamp G, Wood RD 2000 Disruption of the developmentally regulated Rev3l gene causes embryonic lethality. Curr. Biol. 10, 1217–1220. (doi:10.1016/S0960-9822(00)00725-9) 11050392 10.1016/s0960-9822(00)00725-9

[177] Selvaraj A, Prywes R 2003 Megakaryoblastic leukemia-1/2, a transcriptional co-activator of serum response factor, is required for skeletal myogenic differentiation. J. Biol. Chem. 278, 41 977–41 987. (doi:10.1074/jbc.M305679200) 10.1074/jbc.M305679200 14565952

[178] O'Shaughnessy RF, Welti JC, Sully K, Byrne C 2009 Akt-dependent Pp2a activity is required for epidermal barrier formation during late embryonic development. Development 136, 3423–3431. (doi:10.1242/dev.037010) 19762425 10.1242/dev.037010 PMC2752394

[179] Jaenisch R, Bird A 2003 Epigenetic regulation of gene expression: how the genome integrates intrinsic and environmental signals. Nat. Genet. 33, 245–254. (doi:10.1038/ng1089) 12610534 10.1038/ng1089

[180] Gräff J, Kim D, Dobbin MM, Tsai LH 2011 Epigenetic regulation of gene expression in physiological and pathological brain processes. Physiol. Rev. 91, 603–649. (doi:10.1152/physrev.00012.2010) 21527733 10.1152/physrev.00012.2010

[181] Pan J, Jin C, Murata T, Yokoyama KK 2004 Histone modification activities of JDP2 associated with retinoic acid-induced differentiation of F9 cells. Nucleic Acids Symp. Ser. (Oxf). 48, 189–190. (doi:10.1093/nass/48.1.189) 10.1093/nass/48.1.189 17150542

[182] Bowen NJ, Fujita N, Kajita M, Wade PA 2004 Mi-2/NuRD: multiple complexes for many purposes. Biochim. Biophys. Acta 1677, 52–57. (doi:10.1016/j.bbaexp.2003.10.010) 15020045 10.1016/j.bbaexp.2003.10.010

[183] Sugimoto N et al. 2015 Cdt1-binding protein GRWD1 is a novel histone-binding protein that facilitates MCM loading through its influence on chromatin architecture. Nucleic Acids Res. 43, 5898–5911. (doi:10.1093/nar/gkv509) 25990725 10.1093/nar/gkv509 PMC4499137

[184] Mariño-Ramírez L, Kann MG, Shoemaker BA, Landsman D 2005 Histone structure and nucleosome stability. Expert Rev. Proteomics 2, 719–729. (doi:10.1586/14789450.2.5.719) 16209651 10.1586/14789450.2.5.719 PMC1831843

[185] Yap KL, Li S, Muñoz-Cabello AM, Raguz S, Zeng L, Mujtaba S, Gil J, Walsh MJ, Zhou MM 2010 Molecular interplay of the noncoding RNA ANRIL and methylated histone H3 lysine 27 by polycomb CBX7 in transcriptional silencing of INK4a. Mol. Cell. 38, 662–674. (doi:10.1016/j.molcel.2010.03.021) 20541999 10.1016/j.molcel.2010.03.021 PMC2886305

[186] Ikegamii K, Horigome D, Mukai M, Livnat I, MacGregor GR, Setou M 2008 TTLL10 is a protein polyglycylase that can modify nucleosome assembly protein 1. FEBS Lett. 582, 1129–1134. (doi:10.1016/j.febslet.2008.02.079) 18331838 10.1016/j.febslet.2008.02.079 PMC2408875

[187] Marzluff WF, Gongidi P, Woods KR, Jin J, Maltais LJ 2002 The human and mouse replication-dependent histone genes. Genomics 80, 487–498. (doi:10.1006/geno.2002.6850) 12408966

[188] Li Y et al. 2014 AF9 YEATS domain links histone acetylation to DOT1 L-mediated H3K79 methylation. Cell 159, 558–571. (doi:10.1016/j.cell.2014.09.049) 25417107 10.1016/j.cell.2014.09.049 PMC4344132

[189] Iizuka M, Stillman B 1999 Histone acetyltransferase HBO1 interacts with the ORC1 subunit of the human initiator protein. J. Biol. Chem. 274, 23 027–23 034. (doi:10.1074/jbc.274.33.23027) 10.1074/jbc.274.33.23027 10438470

[190] Katoh M, Katoh M 2007 Comparative integromics on JMJD1C gene encoding histone demethylase: conserved POU5F1 binding site elucidating mechanism of JMJD1C expression in undifferentiated ES cells and diffuse-type gastric cancer. Int. J. Oncol. 31, 219–223. (doi:10.3892/ijo.31.1.219) 17549425

[191] Hwa AP, Shyam T, Guozhen W 2012 Shutting down the mobile phone and the downfall of Nepalese society, economy and politics. Pacific Affairs 85, 547–561. (doi:10.5509/2012853547)

[192] Hanno S 2015 Can't we all disagree more constructively? Moral foundations, moral reasoning, and political disagreement. Neuroethics 8, 153–169. (doi:10.1007/s12152-015-9235-6)

[193] Pahwa S, Scoglio C, Scala A 2014 Abruptness of cascade failures in power grids. Sci. Rep. 4, 3694 (doi:10.1038/srep03694) 24424239 10.1038/srep03694 PMC3892437

[194] Mattson MP, Chan SL 2003 Calcium orchestrates apoptosis. Nat. Cell. Biol. 5, 1041–1043. (doi:10.1038/ncb1203-1041) 14647298 10.1038/ncb1203-1041

[195] Lang F, Föller M, Lang KS, Lang PA, Ritter M, Gulbins E, Vereninov A, Huber SM 2005 Ion channels in cell proliferation and apoptotic cell death. J. Membr. Biol. 205, 147–157. (doi:10.1007/s00232-005-0780-5) 16362503 10.1007/s00232-005-0780-5

[196] Tu Y, Stolovitzky G, Klein U 2002 Quantitative noise analysis for gene expression microarray experiments. Proc. Natl Acad. Sci. USA 99, 14031 (doi:10.1073/pnas.222164199) 12388780 10.1073/pnas.222164199 PMC137831

[197] Jackson ES, Wayland MT, Fitzgerald W, Bahn S 2005 A microarray data analysis framework for postmortem tissues. Methods 37, 247–260. (doi:10.1016/j.ymeth.2005.09.006) 16308154 10.1016/j.ymeth.2005.09.006

[198] Barash Y, Dehan E, Krupsky M, Franklin W, Geraci M, Friedman N, Kaminski N 2004 Comparative analysis of algorithms for signal quantitation from oligonucleotide microarrays. Bioinformatics 20, 839–846. (doi:10.1093/bioinformatics/btg487) 14751998 10.1093/bioinformatics/btg487

[199] Seo J, Bakay M, Chen YW, Hilmer S, Shneiderman B, Hoffman EP 2004 Interactively optimizing signal-to-noise ratios in expression profiling: project-specific algorithm selection and detection p-value weighting in Affymetrix microarrays. Bioinformatics 20, 2534–2544. (doi:10.1093/bioinformatics/bth280) 15117752 10.1093/bioinformatics/bth280

[200] Harr B, Schlötterer C 2006 Comparison of algorithms for the analysis of Affymetrix microarray data as evaluated by co-expression of genes in known operons. Nucl. Acids Res. 34, e8 (doi:10.1093/nar/gnj010) 16432259 10.1093/nar/gnj010 PMC1345700

[201] Millenaar FF, Okyere J, May ST, van Zanten M, Voesenek LA, Peeters AJ 2006 How to decide? Different methods of calculating gene expression from short oligonucleotide array data will give different results. BMC Bioinform. 7, 137 (doi:10.1186/1471-2105-7-137) 10.1186/1471-2105-7-137 PMC1431565 16539732

[202] Amend AS, Seifert KA, Bruns TD 2010 Quantifying microbial communities with 454 pyrosequencing: does read abundance count? Mol. Ecol. 19, 5555 (doi:10.1111/j.1365-294X.2010.04898.x) 21050295 10.1111/j.1365-294X.2010.04898.x

[203] Pine PS et al 2016 Evaluation of the External RNA Controls Consortium (ERCC) reference material using a modified Latin square design. BMC Biotechnol. 16, 54 (doi:10.1186/s12896-016-0281-x) 27342544 10.1186/s12896-016-0281-x PMC4921035

[204] GallegoRomero I, Pai AA, Tung J, Gilad Y 2014 RNA-seq: impact of RNA degradation on transcript quantification. BMC Biol. 12, 42 (doi:10.1186/1741-7007-12-42) 24885439 10.1186/1741-7007-12-42 PMC4071332

[205] González-Herrera L, Valenzuela A, Marchal JA, Lorente JA, Villanueva E 2013 Studies on RNA integrity and gene expression in human myocardial tissue, pericardial fluid and blood, and its postmortem stability. Forensic Sci. Int. 232, 218–228. (doi:10.1016/j.forsciint.2013.08.001) 24053884 10.1016/j.forsciint.2013.08.001

[206] Gérard A et al. 2014 Detection of rare antigen-presenting cells through T cell-intrinsic meandering motility, mediated by *Myo1g*. Cell 15, 492–505. (doi:10.1016/j.cell.2014.05.044) 10.1016/j.cell.2014.05.044 PMC4119593 25083865

[207] Müller T, Rumpel E, Hradetzky S, Bollig F, Wegner H, Blumenthal A, Greinacher A, Endlich K, Endlich N 2011 Non-muscle myosin IIA is required for the development of the zebrafish glomerulus. Kidney Int. 80, 1055–1063. (doi:10.1038/ki.2011.256) 21849970 10.1038/ki.2011.256

[208] Coyle RC, Latimer A, Jessen JR 2008 Membrane-type 1 matrix metalloproteinase regulates cell migration during zebrafish gastrulation: evidence for an interaction with non-canonical Wnt signaling. Exp. Cell. Res. 314, 2150–2162. (doi:10.1016/j.yexcr.2008.03.010) 18423448 10.1016/j.yexcr.2008.03.010

[209] Babapulle CJ, Jayasundera NP 1993 Cellular changes and time since death. Med. Sci. Law 33, 213–222. 8366783 10.1177/002580249303300306

[210] Latil M, Rocheteau P, Châtre L, Sanulli S, Mémet S, Ricchetti M, Tajbakhsh S, Chrétien F 2012 Skeletal muscle stem cells adopt a dormant cell state post mortem and retain regenerative capacity. Nat. Commun. 3, 903 (doi:10.1038/ncomms1890) 22692546 10.1038/ncomms1890

[211] Singh M, Ma X, Amoah E, Kannan G 2011 In vitro culture of fibroblast-like cells from postmortem skin of Katahdin sheep stored at 4°C for different time intervals. In Vitro Cell. Dev. Biol. Anim. 47, 290–293. (doi:10.1007/s11626-011-9395-6) 21400020 10.1007/s11626-011-9395-6

[212] Okonkwo C, Singh M 2015 Recovery of fibroblast-like cells from refrigerated goat skin up to 41 d of animal death. In Vitro Cell. Dev. Biol. Anim. 51, 463–469. (doi:10.1007/s11626-014-9856-9) 25539865 10.1007/s11626-014-9856-9

[213] Mahipal HS, Singh M 2014 Recovery of fibroblast-like cells after 160 days of postmortem storage of goat skin tissues in refrigerated media. In Vitro Cell. Dev. Biol. Anim. 50, S40.

[214] Senn P, Oshima K, Teo D, Grimm C, Heller S 2007 Robust postmortem survival of murine vestibular and cochlear stem cells. J. Assoc. Res. Otolaryngol. 8, 194–204. (doi:10.1007/s10162-007-0079-6) 17334849 10.1007/s10162-007-0079-6 PMC2538352

[215] Kauffman SA 1993 The origins of order: self-organization and selection in evolution. New York, NY: Oxford University Press.

[216] Aberg F, Pukkala E, Höckerstedt K, Sankila R, Isoniemi H 2008 Risk of malignant neoplasms after liver transplantation: a population-based study. Liver Transpl. 14, 1428–1436. (doi:10.1002/lt.21475) 18825704 10.1002/lt.21475

[217] Haagsma EB, Hagens VE, Schaapveld M, van den Berg AP, de Vries EG, Klompmaker IJ, Slooff MJ, Jansen PL 2001 Increased cancer risk after liver transplantation: a population-based study. J. Hepatol. 34, 84–91. (doi:10.1016/S0168-8278(00)00077-5) 11211912 10.1016/s0168-8278(00)00077-5

[218] Pozhitkov AE, Neme R, Domazet-Lošo T, Leroux BG, Soni S, Tautz D, Noble PA 2017 Data from: Tracing the dynamics of gene transcripts after organismal death. Dryad Digital Repository. (doi:10.5061/dryad.hv223) 10.1098/rsob.160267 PMC5303275 28123054

