# Supplementary material for: Tracing the dynamics of gene transcripts after organismal death
Source: Open Biol. 2017 Jan 25;7(1):160267. doi: 10.1098/rsob.160267 (PMC5303275; doi:10.1098/rsob.160267)
Supplement: Fig S1; Fig S2; Table S1; Table S2; Table S3 [file rsob160267supp1.docx]

**Electronic Supplementary Information**

Tracing the dynamics of gene transcripts after organismal death

Alex E. Pozhitkov, Rafik Neme, Tomislav Domazet-Lošo, Brian G. Leroux, Shivani Soni, Diethard Tautz and Peter A. Noble^*^.

*Correspondence to: [panoble@washington.edu](mailto:xxxxx@xxxx.xxx)

**This PDF file includes:**

Supplementary Text

Figs S1 and S2

Table S1, S2 and S3

# Supplementary Text

Fig S1. Bioanalyzer results showing total mRNA from the zebrafish.

Fig S2. Bioanalyzer results showing total mRNA from the mouse.

Table S1. Total mRNA extracted (ng/µL tissue extract) from zebrafish by time and replicate sample.

Table S2. Total mRNA extracted (ng/µL tissue extract) from mouse organ/tissue by time and replicate sample.

Table S3. % of global regulator genes and response genes.


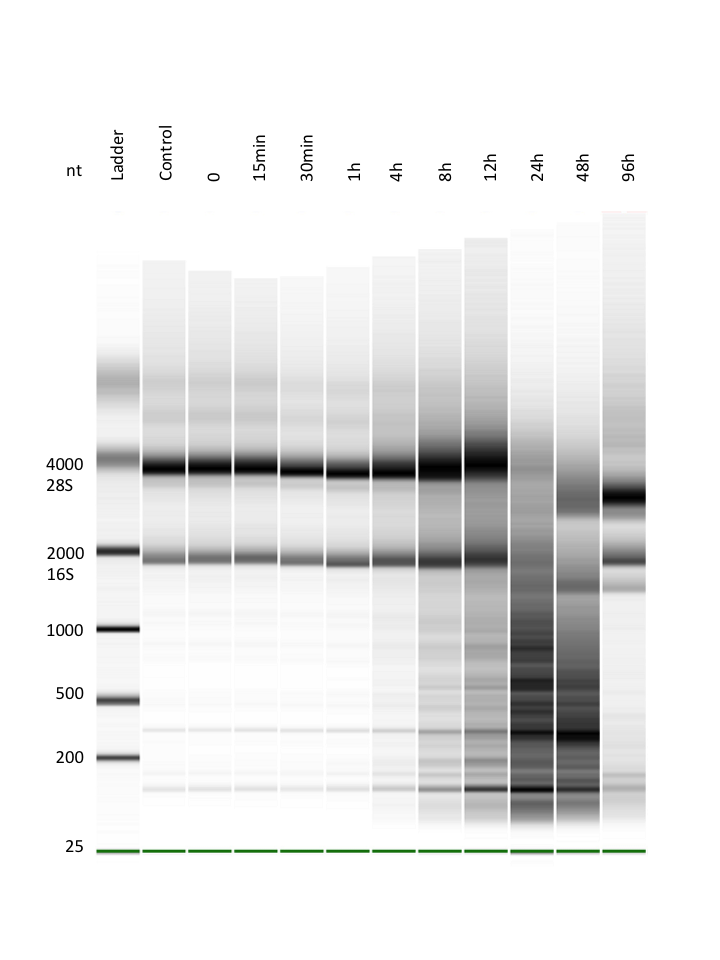


Fig S1. Bioanalyzer results showing total mRNA from the zebrafish. Only one replicate per sampling time is shown. The dominant bands represent the 28S and 18S rRNAs.


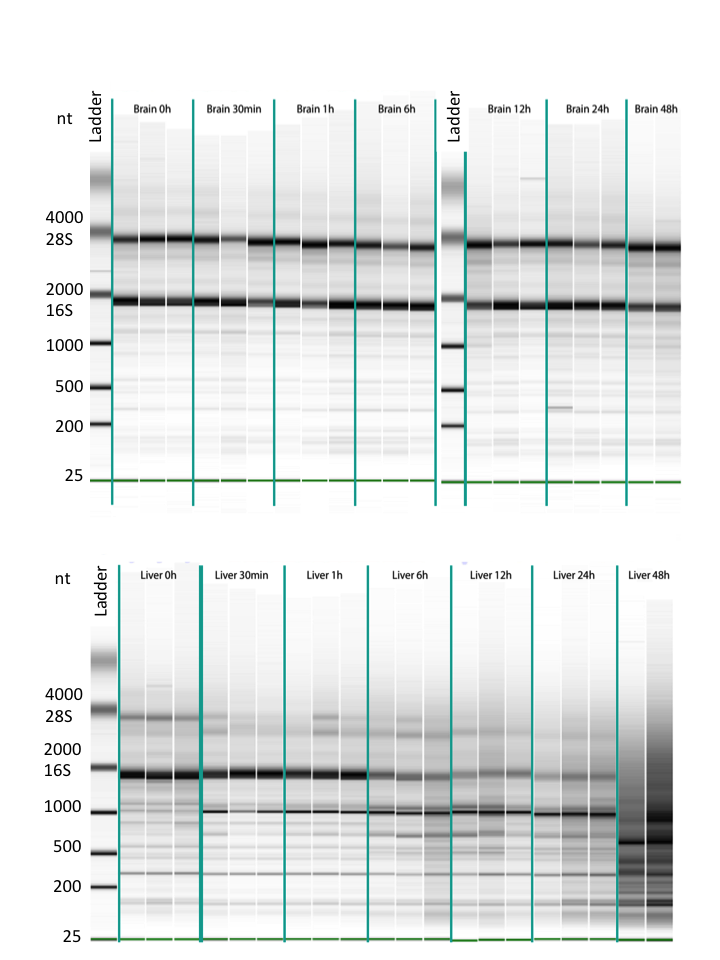


Fig S2. Bioanalyzer results showing total mRNA from the mouse. All replicates per sampling time are shown. The dominant bands represent the 28S and 18S rRNAs.

Table S1. Total mRNA extracted (ng/µL tissue extract) from zebrafish by time and replicate sample.

| Time (h) | Repl#1 | Repl#2 |
| --- | --- | --- |
| Live | 1606 | 1648 |
| 0 | 1632 | 1481 |
| 0.25 | 1768 | 1573 |
| 0.5 | 1457 | 1481 |
| 1 | 1864 | 1514 |
| 4 | 1251 | 1651 |
| 8 | 1605 | 1499 |
| 12 | 1365 | 1422 |
| 24 | 1087 | 539 |
| 48 | 339 | 428 |
| 96 | 183 | 183 |

a, no replicate taken

Table S2. Total mRNA extracted (ng/µL tissue extract) from mouse organ/tissue by time and replicate sample.

| Organ | Time | Repl#1 | Repl#2 | Repl#3 |
| --- | --- | --- | --- | --- |
| Liver | 0 | 432 | 387 | 443 |
| Liver | 0.5 | 533 | 651 | 569 |
| Liver | 1 | 421 | 426 | 601 |
| Liver | 6 | 541 | 624 | 528 |
| Liver | 12 | 839 | 450 | 845 |
| Liver | 24 | 1021 | 510 | 1066 |
| Liver | 48 | 1453 | 1197 | -^a^ |
|  |  |  |  |  |
| Brain | 0 | 169 | 226 | 210 |
| Brain | 0.5 | 174 | 166 | 410 |
| Brain | 1 | 194 | 485 | 264 |
| Brain | 6 | 401 | 269 | 256 |
| Brain | 12 | 379 | 258 | 203 |
| Brain | 24 | 249 | 324 | 400 |
| Brain | 48 | 397 | 310 | - |

a, no replicate taken

Table S3. % of global regulator genes and response genes. Approx. 33% of all transcripts with increased abundance involved global regulation genes.

| **Global gene regulators and other response genes** | **Zebrafish** | **Mouse** | **Combined** |
| --- | --- | --- | --- |
| Transcription factors and transcriptional regulatory genes | 17 | 12 | 14 |
| Cell signaling genes | 24 | 16 | 19 |
| Other response genes | 59 | 73 | 67 |
